# Zinc-finger protein CNBP alters the 3-D structure of lncRNA *Braveheart* in solution

**DOI:** 10.1038/s41467-019-13942-4

**Published:** 2020-01-09

**Authors:** Doo Nam Kim, Bernhard C. Thiel, Tyler Mrozowich, Scott P. Hennelly, Ivo L. Hofacker, Trushar R. Patel, Karissa Y. Sanbonmatsu

**Affiliations:** 1 Theoretical Biology and Biophysics Group, Los Alamos National Laboratory, Los Alamos, New Mexico USA; 2 Department of Theoretical Chemistry, University of Vienna, Vienna, Austria; 3 Alberta RNA Research & Training Institute, Department of Chemistry and Biochemistry, University of Lethbridge, Lethbridge, Alberta Canada; 4 New Mexico Consortium, Los Alamos, New Mexico USA; 5 Bioinformatics and Computational Biology, Faculty of Computer Science, University of Vienna, Vienna, Austria; 6 Cumming School of Medicine, University of Calgary, Calgary, Alberta Canada; 7 Li Ka Shing Institute of Virology, University of Alberta, Edmonton, Alberta Canada

**Keywords:** Biophysical chemistry, RNA, Biophysics, Computational biophysics, Molecular biophysics

## Abstract

Long non-coding RNAs (lncRNAs) constitute a significant fraction of the transcriptome, playing important roles in development and disease. However, our understanding of structure-function relationships for this emerging class of RNAs has been limited to secondary structures. Here, we report the 3-D atomistic structural study of epigenetic lncRNA, *Braveheart (Bvht)*, and its complex with CNBP (Cellular Nucleic acid Binding Protein). Using small angle X-ray scattering (SAXS), we elucidate the ensemble of *Bvht* RNA conformations in solution, revealing that *Bvht* lncRNA has a well-defined, albeit flexible 3-D structure that is remodeled upon CNBP binding. Our study suggests that CNBP binding requires multiple domains of *Bvht* and the *RHT/AGIL* RNA motif. We show that RHT/AGIL, previously shown to interact with CNBP, contains a highly flexible loop surrounded by more ordered helices. As one of the largest RNA-only 3-D studies, the work lays the foundation for future structural studies of lncRNA-protein complexes.

## Abstract (web summary)

Many RNA systems possess highly ordered 3-D structures that are essential to their function. Here the authors demonstrate that the long non-coding RNA *Braveheart* possesses a flexible but defined 3-D structure which is remodeled upon binding the protein CNBP.

## Introduction

Long non-coding RNAs (lncRNAs) are emerging as key players in a variety of biological processes, including gene expression, genetic imprinting, histone modification, and chromatin dynamics^1^. To perform these crucial functions, they interact with proteins, DNA and other RNAs. An understanding of lncRNA-ligand interaction using biochemical and biophysical methods is essential to elucidate the mechanism by which the lncRNAs execute their functions^2^. However, there are a number of challenges associated with biophysical studies of lncRNAs. Despite the impressive number of annotated transcripts^2^, only a small number of lncRNAs (e.g., *Braveheart*^3^, *COOLAIR*^4^, *HOTAIR*^5^, *ROX1*^6^, *ROX2*^6^, *SRA*^7^, *XIST repeat A*^8,9^, *TancRNA*^10^, and 3′ end of human, zebrafish, and lizard *MALAT1*^10^) have undergone secondary structure analysis via chemical probing (e.g., selective 2′ hydroxyl acylation analyzed by primer extension, i.e., SHAPE or dimethyl sulfate, i.e., DMS) or low-resolution enzymatic probing^2^. Akin to early studies of the ribosome, these secondary structures provide the framework for understanding structure–function relationships, producing valuable information like local modularity; however, chemical probing studies yield little information about the overall 3-dimensional (3-D) structure^11^. As their name suggests, long noncoding RNAs are often large, making their preparation and purification for in vitro studies very challenging. In addition, longer lengths make fold determination more challenging. The lncRNAs are also typically much less abundant than messenger RNAs. For example, only a few copies per cell of immune-gene priming lncRNAs are expressed^12^. Moreover, lncRNAs usually have a very short half-life (less than 9–12 h, with the exception of *MALAT1*^13^) making in vivo studies challenging as well. Finally, the large RNA molecules often have flexible regions that further pose challenges for RNA structure determination^14^.

Although 3-D structures are often essential to establish structure–function relationships, no such studies have been performed for intact, epigenetic long noncoding RNAs, to our knowledge. In fact, a widely held perspective in the RNA community is that lncRNAs tend to be too flexible and unstable for nuclear magnetic resonance (NMR) and crystallization studies. Interestingly, many biologically important RNAs have dynamically changing conformations, making structure determination challenging^2,15^. However, it has been shown, using chemical probing, that several lncRNAs and portions of lncRNAs adopt well-organized, modular secondary structures^3–5,7,9^. Furthermore, because SHAPE probing reports on the physical mobility of the backbone for each nucleotide, the many regions of low-SHAPE reactivity in these lncRNA systems demonstrate that these RNAs possess regions with well-defined secondary and possible tertiary interactions^16^. In addition, when we performed SHAPE and DMS probing of *Bvht*, reactivities were fairly similar regardless of the probing method^3^. Likewise, we obtained similar reactivities of probing for the 3′ end of *MALAT1*, whether we used SHAPE or DMS^10^. When we probed the steroid receptor RNA activator (SRA), derived secondary structures were fairly similar to each other whether we used SHAPE, DMS, in-line or RNase V1^7^. For *COOLAIR*, reactivities from both SHAPE and CMCT (1-cyclohexyl-3-(2-morpholi-noethyl) carbodiimide metho-p-toluene sulfonate) are similar to each other^4^.

These probing data indicate that at least these particular lncRNAs do possess well-organized secondary structures. If these lncRNAs were intrinsically disordered, probing data would have yielded a superposition of all potential structures with prominent protection patterns. The Pyle group also confirmed this phenomenon. For example, DMS and terbium reactivities showed 92–93% agreement with SHAPE data for *HOTAIR*^5^. DMS and SHAPE showed similar reactivities for *RepA* as well^9^. Building on chemical probing data, the next step is to investigate the 3-D structure of these lncRNAs. One of the well-known lncRNAs, *Braveheart* (*Bvht*), binds to cellular nucleic acid binding protein (CNBP) and the *SUZ12* component of the *PRC2* complex altering chromatin modification^17^, affecting the expression of many genes that are important for cardiovascular lineage commitment, such as *MesP1*, *GATA4*, *HAND1*, *HAND2*, *NKX2.5*, and *TBX5*^2^. While its secondary structure has been studied^3^, the 3-D structure of *Bvht* is unknown.

Regarding 3-D methods in structure determination, high-resolution structure determination methods have several challenges with long RNA molecules and complexes with their interacting partners^14^. For example, while many excellent NMR studies of biomolecules have been performed^18–22^, this method typically has been limited to proteins smaller than 50 kDa^23^ and RNAs smaller than 100–300 nucleotides^24^. In addition, crystallographic studies of RNA molecules are typically more challenging than DNA and proteins. In particular, as we show in this study, the full-length *Bvht* lncRNA has a well-defined 3-D structure, but has, at the same time, flexible regions, making it very challenging to trap the molecule in a single conformation, as is required for X-ray crystallography. Of course, there are many X-ray crystallography-based RNA structures. However, except for the ribosome, group I intron and group II intron^25–27^, these RNAs are relatively small and highly ordered.

On the other hand, small angle X-ray scattering (SAXS) is an excellent alternative method that allows structural studies of fully or partially unfolded proteins and RNAs without being limited by molecular mass^14^. Therefore, solution scattering is often employed for systems that do not readily crystallize^28^. SAXS can access large dynamic motions as well. There are several RNA^29^ and RNA–protein complex structures that have been determined by SAXS^14^.

To address the above-mentioned need for in-depth structural studies of *Bvht*, we performed extensive biophysical experiments and computational studies to investigate the three-dimensional structure, primarily based on SAXS. We note that many excellent modeling pipelines have been used for 3-D RNA structure determination^30,31^. Below, we present a modeling pipeline which is particularly useful for SAXS studies of very large RNA systems. Using this modeling pipeline, we are able to show the 3-D structure of a full lncRNA, *Bvht*. We note that a partial structure of a lncRNA was reported, e.g., 65 nucleotide long *MALAT1* ENE (expression and nuclear retention element) and A-rich tract^32^. In addition, we show the 3-D structure of *Bvht*-CNBP complexes. CNBP, a zinc-finger transcription factor, binds *Bvht* and is important for heart cell lineage differentiation^3^. This structural study of a lncRNA–protein complex will be informative for further studies, such as the dynamic association between RNA and RNA-binding proteins, a process important in many aspects of the life-cycle of lncRNAs, including their processing, modification, stability, and localization^33^.

## Results

### Effect of Mg^2+^ on the solution structure of *Bvht*

**Fig. 1:**
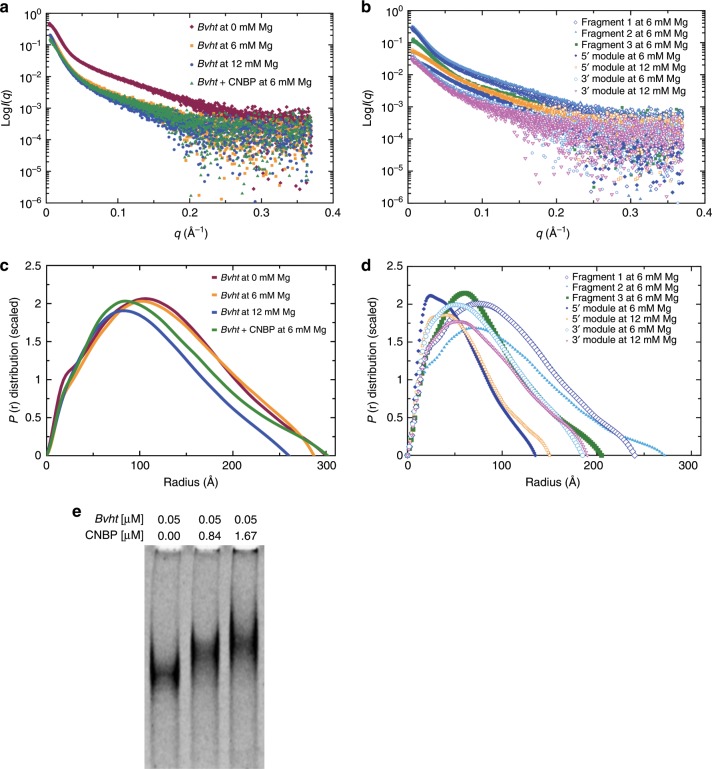
SAXS and EMSA data for lncRNA *Bvht*. **a** Scattering intensity vs. scattering angle (*q* = 4*π*sin*θ*/*λ*), indicating the dependence on Mg^2+^ concentration and effect of *Bvht-*CNBP complex formation. **b** Scattering intensity vs. scattering angle for isolated subregions of *Bvht* lncRNA. **c** Pair-distance distribution function for full-length *Bvht* at various Mg^2+^ concentrations and for *Bvht*-CNBP complex. **d** Pair-distance distribution function for subregions of *Bvht*. **e** EMSA for full-length *Bvht* with increasing CNBP concentrations.

**Fig. 2:**
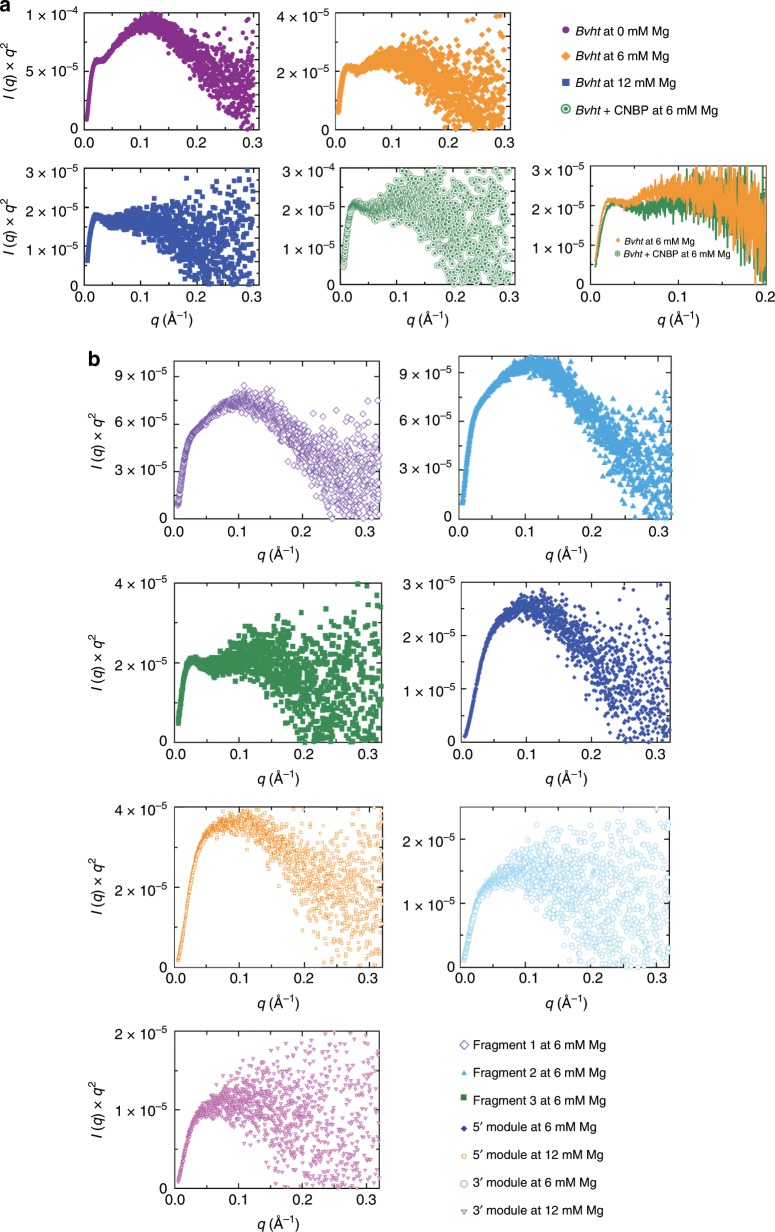
Kratky plots of *Bvht*. **a** Data of the full-length *Bvht* at various Mg^2+^ concentrations and of the *Bvht*-CNBP complex show that overall, all RNA molecules are folded. Upon binding with CNBP, full-length *Bvht* undergoes a conformation change (bottom right). **b** Data of *Bvht* subregions.

Salt-dependent association can be critical for biological function^34^. In RNA polymers, divalent magnesium (Mg^2+^) is essential for folding, higher-order interactions and function^35^. As such, sensitivity to Mg^2+^ is an indicator of the presence of tertiary interactions. In fact, due to its small ionic radius, Mg^2+^ has the highest charge density from all ions in cells^36^ and is more influential than potassium (K^+^) for RNA conformation^37^. Therefore, we determined the effect of Mg^2+^ concentration on the 3-D solution conformation of *Bvht*. We collected SAXS data using a size exclusion chromatography device connected in-line with the SAXS instrument (to separate any aggregated/degraded RNA material) for *Bvht* in 0, 6, and 12 mM MgCl_2_. We selected data from a monodispersed SEC-SAXS peak and merged them as discussed below (Methods section). The merged SEC-SAXS data along with EMSA data are presented in Fig. 1. The buffer-subtracted merged data were then first analyzed by the Guinier method (plot of (*I*(*q*)) vs. (*q*^2^)), which allows detection of homogeneity and determination of the radius of gyration (*R*_g_) based on the data from the low angle region^38^. The Guinier plots presented in Supplementary Fig. 1 display linearity for small *q* values, suggesting that *Bvht* samples are aggregation free. Next, we performed Kratky analysis (plot of *I*(*q*)*q*^2^ vs. *q*) of SAXS data that allows examination of the folding state of biomolecules^39^. For example, globular biomolecules will display a bell-shaped distribution. The Kratky plots for *Bvht* samples under investigation (Fig. 2) suggest that the samples are folded.

It was previously reported that often the large lncRNAs adopt more compact structures with increasing Mg^2+^ concentration^5,9^. In order to determine whether Mg^2+^ has any effect on *Bvht* conformation in solution, we performed an indirect Fourier transformation to convert the reciprocal-space information of ln(*I*(*q*)) vs. q into the real space electron pair-distance distribution function (*P*(*r*)) to obtain reliable values of *R*_g_ and *D*_max_ (radius at which *P*(*r*) approaches to zero) for *Bvht* samples using the program *GNOM*. The benefit of using this method over Guinier analysis for *R*_g_ determination is that the *P*(*r*) method utilizes the entire dataset, whereas the Guinier method that is restricted to the data in the low*-q* region. Consistent with this observation, the SEC-SAXS data for *Bvht* suggested that the full-length *Bvht* becomes more compact as Mg^2+^ concentration increases from 0 to 12 mM (Table 1). For example, the maximum particle dimension (*D*_max_) for *Bvht* solubilized in the absence of Mg^2+^ was 300 Å, which decreases to 287 and 260 Å in the presence of 6 and 12 mM MgCl_2_, respectively. Visual inspection also indicates that *Bvht* conformations tend to be more compact as we increase Mg^2+^ concentration (Supplementary Fig. 2). This may result from a combination of Mg^2+^ effects, including increased electrostatic screening, outer sphere coupling Mg^2+^-RNA interactions and/or specific chelation of Mg^2+^ by the RNA^40–42^.

**Table 1:** Dependence of structural characteristics of full-length *Bvht* (636 nucleotides, 206 kDa) on Mg^2+^ concentration.

Mg^2+^ concentration	0 mM	6 mM	12 mM
*R*_g_ (Å)	98.1 ± 0.2	99.53 ± 0.2	84.8 ± 0.7
*D*_max_ (Å)	300	287	260
*χ*^2^	1.3	1.28	1.18
NSD	1.27 ± 0.02	1.21 ± 0.04	1.24 ± 0.04
Resolution (Å)	13.9–18.6	13.6–35.0	14.2–37.2

*R*_g_ was determined using the *P*(*r*) function that utilizes both lower and higher angle datasets for each sample. *D*_max_ is the radius where the *P*(*r*) distribution approaches to zero. The *χ*^2^ value indicate the goodness of fit between the experimentally collected and model-derived SAXS data, whereas the NSD (normalized spatial discrepancy) describes an agreement between individual models for each dataset. The resolution of models was calculated by *phenix.mtriage*^63^. See Methods section for additional detail

Mg^2+^ dependent conformational changes are more obvious for larger lncRNAs (e.g., 206 kDa for our full-length *Bvht*^3^ or up to 700 kDa for other RNAs^5,9^). For smaller lncRNAs (e.g., 30–69 kDa for *Bvht* modules), Mg^2+^ dependent conformational changes are more difficult to observe (Supplementary Fig. 3). For example, when we measured SHAPE reactivities of the 3′ end of human, zebrafish and lizard *MALAT1* (46 kDa) at 0 and 6 mM Mg^2+^, they showed small differences in signal^10^.

### Structures of sub-domains support full-length structure

In addition to *Bvht*^3^, other lncRNAs such as *COOLAIR*^4^, *HOTAIR*^5^, *SRA*^7^, and *XIST repeat A* (*RepA*)^9^ fold modularly. Here, small sections of each lncRNA (“modules”) possess secondary structures that fold independently within the lncRNA. Many other lncRNAs likely fold in a modular fashion. This trend seems to enable each lncRNA to contain distinct functional structures. For example, *HOTAIR* possesses distinct binding domains for *PRC2* and *LSD1* complexes, making it modular bifunctional RNA^43^. Modularity in structure would also aid in cotranscriptional recruitment of epigenetic factors to chromatin^44^, thought to play an important role lncRNA–chromatin interactions. We have identified several modular folds in *Bvht* by probing the secondary structure of various subregions and comparing the profiles with the lncRNA as a whole. As such, we hypothesized that SAXS-based structures for each module can be determined independently from the whole lncRNA.

**Fig. 3:**
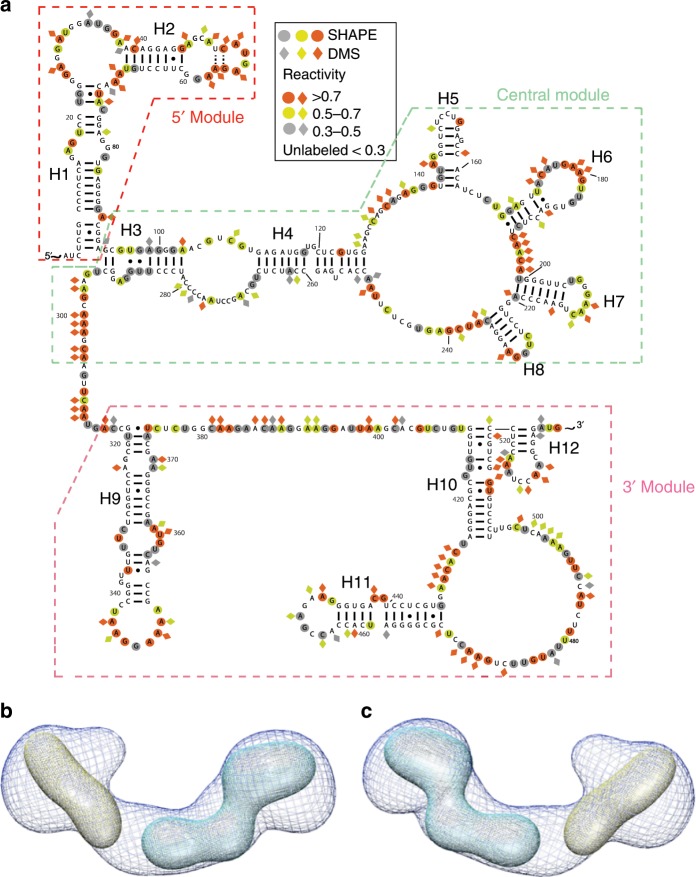
Structural studies of *Bvht* modular sub-domains at 6 mM Mg^2+^. **a** Secondary structures of full-length *Bvht* based on chemical probing experiments, depicting its modular sub-domains. **b** Averaged 3-D SAXS solution conformation of full-length *Bvht* and its modular sub-domains. Blue mesh, averaged solution structure of full-length *Bvht*; yellow, 5′ module of *Bvht*; cyan, 3′ module of *Bvht*. While multiple orientations of the modules fit into the full-length map, we chose orientations most consistent with the connectivity of the secondary structure. **c** 180° rotated view of (**b**).

As a positive control to test this hypothesis, we measured SEC-SAXS profiles of modular sub-domains of *Bvht* (98–224 nts in length) that do not overlap with each other and have modular secondary structures (Fig. 3 and Table 2). The central module (nucleotides # 87–305) did not display mono-dispersity during SEC-SAXS. Therefore, we did not process the data. However, we were able to fit the low-resolution structures for 5′ and 3′ modules of *Bvht* to the full-length *Bvht* structure, suggesting that the individual secondary structures of these two modules are consistent with their secondary structures in full-length *Bvht*.

**Table 2:** Structural characteristics of independent *Bvht* modules.

Sample^a^	5′ module		3′ module		Full-length	
MW (kDa)	30.4		68.6		206	
MgCl_2_ concentration	6 mM	12 mM	6 mM	12 mM	6 mM	12 mM
*R*_g_ (Å)	42 ± 0.2	46 ± 0.2	61 ± 0.3	63 ± 0.4	99.53 ± 0.2	85 ± 0.7
*D*_max_ (Å)	135	150	185	190	287	260
*χ*^2^	1.2	1.2	1.2	1.2	1.3	1.18
NSD	0.85 ± 0.03	0.88 ± 0.02	1.06 ± 0.02	1.04 ± 0.03	1.21 ± 0.04	1.24 ± 0.04
Resolution (Å)	31.3	31.8	34.9–36.5	34.1–36.0	13.6–35.0	14.2–37.2

^a^Central module (nucleotides # 87–305 with expected MW of 67.3 kDa) sample did not show mono-dispersity. Therefore, we did not process the data

5′ module encompasses nucleotides # 1–98, 3′ module encompasses nucleotides # 315–538, full-length encompasses nucleotides GG and # 1–634. See Table 1 for more information on SAXS data analysis

**Fig. 4:**
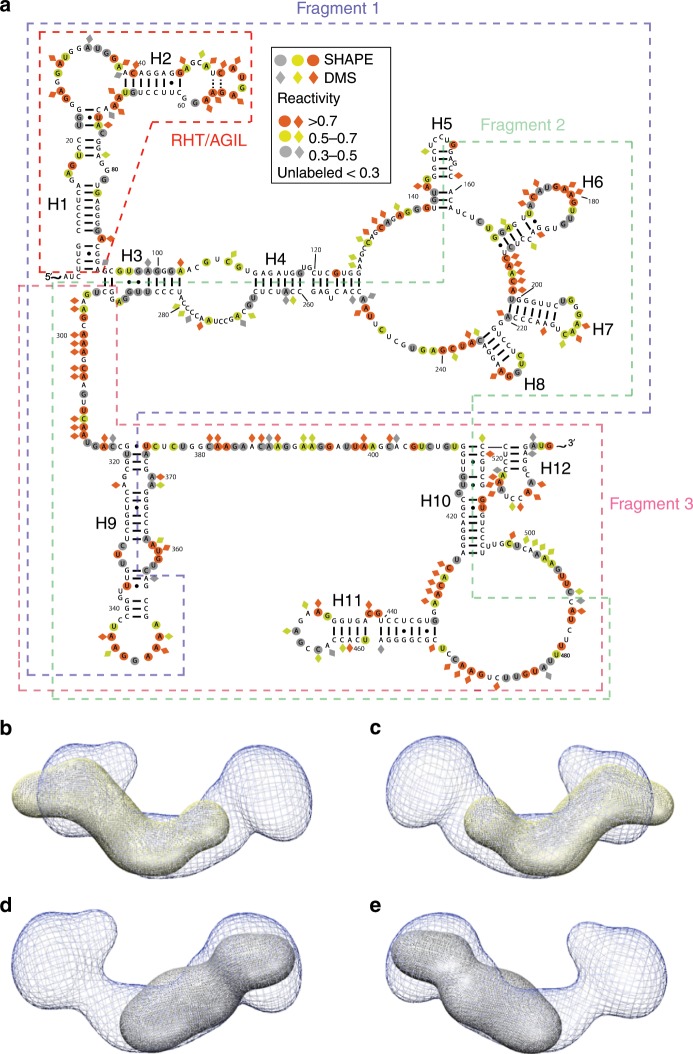
Structural studies of *Bvht* overlapping fragments at 6 mM Mg^2+^. **a** Secondary structures of full-length *Bvht* and its overlapping fragments. **b**–**e** Averaged 3-D SAXS solution conformation of full length *Bvht* and its overlapping fragments. **b** Blue mesh, averaged solution structure of full-length *Bvht*; yellow fragment 1 of *Bvht*. **c** 180° rotated view of (**b**). **d** Gray, fragment 3 of *Bvht*. **e** 180° rotated view of (**d**).

As a negative control, we measured SEC-SAXS profiles of overlapping fragments (344–358 nts in length) of *Bvht* (Fig. 4 and Table 3). Some of these fragments split helical elements and do not have modular secondary structures (i.e., they do not contain both sides of an RNA double helix). For example, fragment 1 splits helix H9, while fragment 2 splits helices H3, H4, H5 and H10. Therefore, we do not necessarily expect the structures of these fragments to be directly related to the structure, or portions of the structure of the intact, full-length *Bvht* lncRNA. We find the solution structure of fragment 3 fits nicely into the full-length *Bvht* solution structure, consistent with the fact that the fragment 3 does contain a modular secondary structure. In the case of fragment 1, the fit is poor, presumably since it only contains approximately half of H9, which may alter the fold relative to the intact *Bvht* RNA molecule. Fragment 2 contains four split helices. Thus, we expect it to have a dramatically different fold relative to the intact *Bvht* RNA. Among ~230 total nucleotides, about 38% of it (e.g., ~8 5′ terminal nucleotides and ~80 3′ terminal nucleotides) cannot form base-pairs found in the full-length *Bvht*.

**Table 3:** Structural characteristics of *Bvht* fragments at 6 mM Mg^2+^.

Sample	Fragment 1	Fragment 2	Fragment 3	Full-length
MW (kDa)	116	113	111	206
*R*_g_ (Å)	78.4 ± 0.2	81.7 ± 0.7	63.9 ± 0.2	99.53 ± 0.2
*D*_max_ (Å)	240	271	205	287
*χ*^2^	1.7	1.4	1.4	1.3
NSD	1.27 ± 0.03	1.12 ± 0.02	1.02 ± 0.02	1.21 ± 0.04
Resolution (Å)	34.1–38.5	34.5	36.7	13.6–35.0

Fragment 1 encompasses nucleotides # 1–358, fragment 2 encompasses nucleotides # 156–504, Fragment 3

encompasses nucleotides # 291–588 and # 589−634 is 3′ structure cassette for primer extension after SHAPE and DMS probing. See Table 1 for more information on SAXS data analysis

The pair-distance distribution function (*P*(*r*)) plots presented in Fig. 1d have skewed bell shape curves with extended tails, indicating that these *Bvht* fragments generally adopt extended structures. For example, *Bvht* fragments (MW ~111–116 kDa) have an *R*_g_ of ~64–82 Å and *D*_max_ of ~205–271 Å. In contrast, the full-length *Bvht* (206 kDa MW) has an *R*_g_ of ~85–99 Å and *D*_max_ of ~260–300 Å. These results suggest that each fragment tends to extend flexibly rather than collapsing in a globular fashion.

We also found that fragment 3 of *Bvht* is significantly more compact than other fragments. Fragments 1 and 2 were quite similar to each other in terms of their solution parameters (*R*_g_, *D*_max)_). However, fragment 3 has a smaller volume than these two other fragments (Table 3). This shows that *Bvht* fragments do not have a monotonic relationship between the volume and nucleotide length unlike a series of riboswitches^34^. Chen et al. previously merged SAXS-based *R*_g_ values and reported the monotonic relationship in different riboswitches (e.g., *R*_g_ values increase fairly linearly with increasing nucleotide length)^34^.

### Ensemble of 3-D models is consistent with SAXS data

**Fig. 5:**
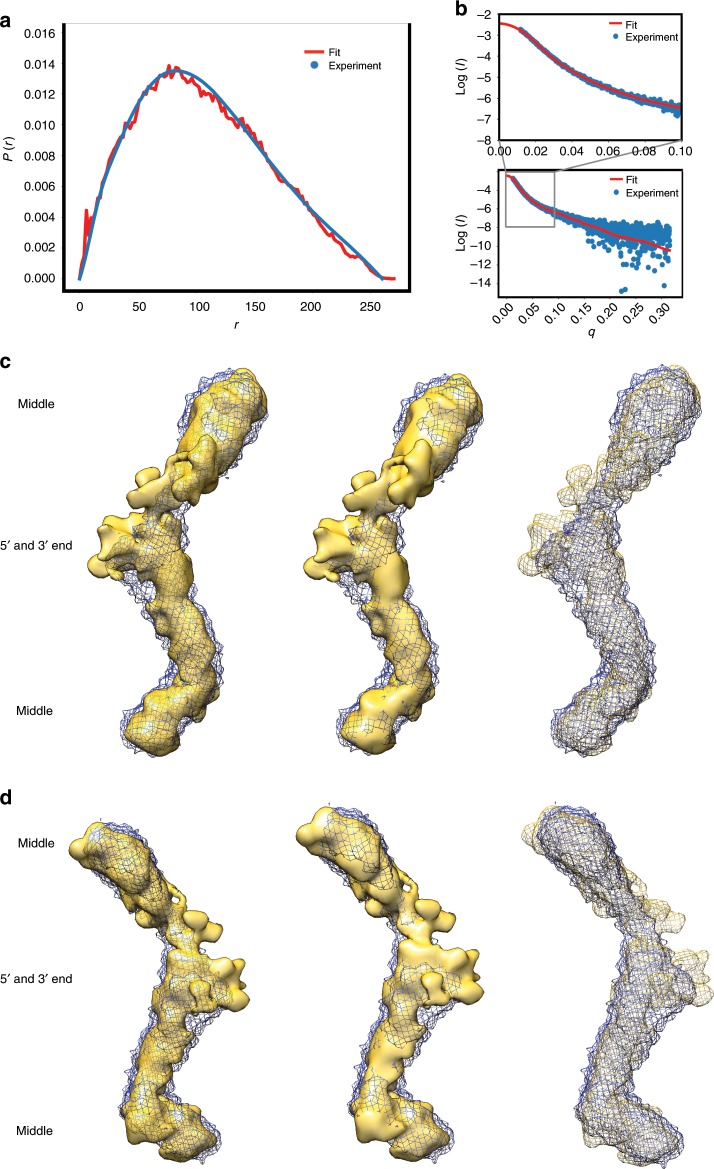
Ensemble of atomistic models of *Bvht* matches measured SAXS data of *Bvht* at 12 mM Mg^2+^. **a** Pair-distance distribution function displays agreement between experimental data (blue) and data back-calculated from computational models (red). **b** Scattering intensity vs. angle indicates agreement between experimental and model derived data. **c** A comparison of averaged solution structures of full-length *Bvht* derived from SAXS data (meshed blue) and an ensemble of computationally derived atomistic models (yellow) shows agreement (Supplementary Fig. 6 and Supplementary Fig. 7). SAXS measured structure was rendered with two different surface opacities and with wire mesh to clearly display surfaces of both SAXS measured solution structure and solution structure of ensemble atomistic model (left, low opacity; middle, high opacity; right, yellow wire mesh). **d** 180° view of (**c**). See Supplementary Movie 1 for 360° rotation.

**Fig. 6:**
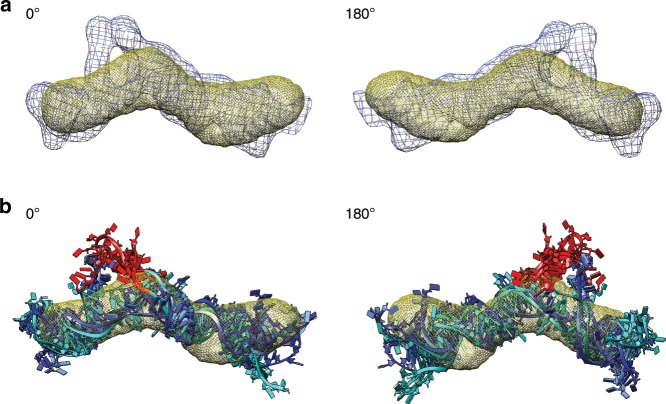
Superposition of atomistic models of *Bvht* 5′ module with experimental structure (yellow mesh). **a** Ensemble of simulated structures of the five top *χ* ranked atomistic models of *Bvht* 5′ module (blue mesh) superimposed with SAXS structure (yellow mesh). **b** Five top *χ* ranked atomistic models of *Bvht* 5′ module at 6 mM Mg^2+^ (cyan to dark blue gradient). As in the full-length *Bvht*, atoms outside of SAXS-derived solution structure may indicate flexible regions. Most of this “flexible” region is the 11-nucleotide region (colored red) in the *RHT/AGIL* motif known to be essential to bind CNBP^3^. See Supplementary Movie 3 for more information.

Modeling SAXS data with atomistic structures presents a different set of challenges relative to crystallography as we can only obtain low-resolution structural information from solution scattering data (for our cases, 13.4–38.5 Å resolution). Using the RNA modeling program, *ERNWIN*^45^, we have produced an ensemble of atomistic RNA structures highly consistent with our SAXS data (Fig. 5, Supplementary Fig. 4 and Supplementary Fig. 5). We also used *Bvht* secondary structure information as restraints (Figs. 3 and 4). We modeled full-length *Bvht* atomistic models (Supplementary Movie 1, Supplementary Fig. 6 and Supplementary Fig. 7) and selected an ensemble of models that fit with the pair-distance distribution function, and with raw scattering data (with *χ* values of 1.7–2.6, and an average value of 2.1), consistent with our former SAXS based computational approach^29^. When superimposing 30 top-ranked models, identifying the most densely populated regions, and comparing this to the SAXS-derived solution structures, we find that the computational structures closely match with the SAXS-derived low-resolution solution structures (Fig. 5). The close agreement not only gives us confidence in our 3-D models, but also in our 2-D secondary structure, upon which the 3-D models are based. In addition, using *ERNWIN*, we modeled atomistic structures of the 5′ module of *Bvht* (Fig. 6), which also agree well with the pair-distance distribution function. The simulated annealing model building approach from SAXS data suggests that *Bvht* has flexible regions, leading to minor variation in each of the low-resolution atomistic structures we calculated. To account for this intrinsic flexibility, we optimized the pair-distance distribution of the ensemble as opposed to individual structures during structure prediction, sampling multiple individual trajectories.

### CNBP binding requires multiple structural domains of *Bvht*

**Fig. 7:**
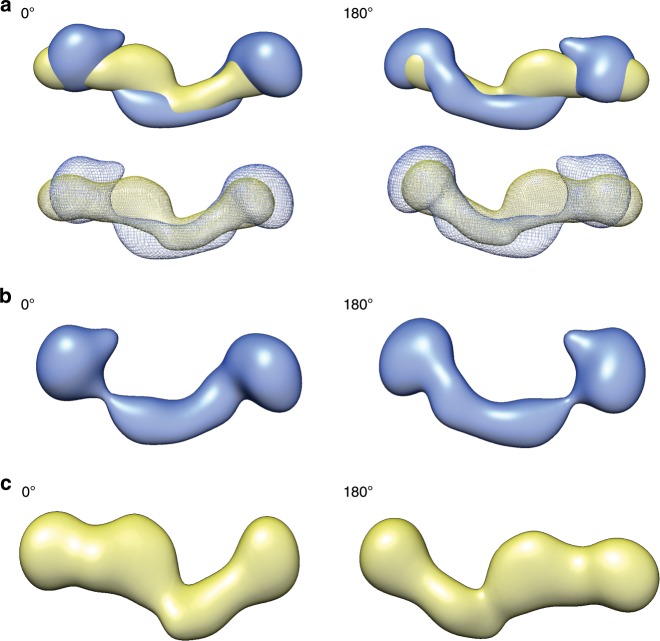
Solution conformation of *Bvht* and its complex with CNBP. **a** Blue, averaged solution structure of *Bvht* only; yellow, averaged solution structure of *Bvht-*CNBP complex. **b** *Bvht* only. **c** *Bvht*-CNBP complex. Individual solution structures are presented in Supplementary Fig. 9.

CRISPR/Cas9 genome editing studies demonstrated that the right-hand-turn (*RHT*)/5′ asymmetric G-rich internal loop (*AGIL*) motif in *Bvht* is essential for cardiovascular lineage commitment. Interestingly, CNBP (a zinc-finger transcription factor) antagonizes this function by binding to the *AGIL* motif of *Bvht*^3^. Accordingly, we were curious to know if binding with CNBP affects *Bvht* conformation. Therefore, we performed SAXS with the CNBP sample as well. The Guinier plot presented in Supplementary Fig. 8 displays linearity for small *q* values suggesting that CNBP samples are aggregation free. Although it is relatively small, the increase in *D*_max_ for the protein–RNA system relative to *Bvht* RNA alone indicates that *Bvht* and CNBP formed a complex (Table 4). In addition, we observed by EMSA that *Bvht* migrates more slowly with higher CNBP concentrations (Fig. 1e). While we are hesitant to overinterpret the SAXS results due to dynamic conformational changes and low-resolution of SAXS data, we observed that CNBP binding is more evident with full-length *Bvht* than its fragments/modules (Fig. 7, Supplementary Fig. 9 and Supplementary Fig. 10). We believe that this small structural difference by CNBP binding results from weak interaction between fragment 1 of *Bvht* and CNBP. Fragments 2 and 3 of *Bvht* also showed weak interaction with CNBP (Supplementary Table 1).

**Table 4:** Dependence of *Bvht* structures on CNBP protein (22 kDa) binding (6 mM Mg^2+^).

Sample	Fragment 1 of *Bvht*		Full-length *Bvht*	
CNBP presence	−CNBP	+CNBP	−CNBP	+CNBP
MW (kDa)	116	138	206	228
R_g_ (Å)	78.4 ± 0.2	82.4 ± 0.5	99.53 ± 0.2	95.1 ± 0.4
D_max_ (Å)	240	270	287	301
*χ*^2^	1.15	1.7	1.3	1.3
NSD	1.20 ± 0.03	1.14 ± 0.04	1.21 ± 0.04	1.29 ± 0.03
Resolution (Å)	34.1–38.5	32.1	13.6–35.0	13.4–17.7

See Table 1 for more information on SAXS data analysis

Our data also suggests that CNBP facilitates compaction of the full-length *Bvht* RNA (e.g., *R*_g_ in Table 4, Fig. 1). Based on pair-distance distribution analysis, we confirmed that upon interaction with CNBP, *Bvht* undergoes conformational changes. This compaction due to CNBP-binding is consistent with overall compaction observed in previous riboswitch studies (e.g., ligand-bound riboswitches are generally more compact than the free riboswitches^34^). Interestingly, the compaction of *Bvht* by CNBP and the EMSA binding results, when taken together with the module analysis, suggest that CNBP interacts with at least two distinct sites on the *Bvht* RNA, which, in turn, lead to a more compact state.

In summary, as we compare the low-resolution structures of *Bvht* only and *Bvht*-CNBP complex, we observed an obvious shape change for the full-length *Bvht*-CNBP complex, while the fragment 1 RNA–CNBP complex did not show a significant difference relative to the fragment 1 only solution structure, suggesting that intact, full-length *Bvht* is required for efficient CNBP binding.

## Discussion

In the epigenetics and pharmaceutical communities, there has been great interest in the question: do lncRNAs have well-defined structures? It has not been clear whether the majority of lncRNAs are disordered, extended or compact^46^. Due to their large structures (200–100,000 nts) and dynamic binding function with partner molecules (including proteins), it is sometimes assumed that lncRNA structures are generally too disordered, or highly flexible to be studied by high-resolution structural determination methods. However, based on secondary structure determination for nine different lncRNAs, it is evident that these lncRNAs do contain modular structures. In fact, regarding RNA systems in general, there are other RNAs of similar sizes (e.g., group I and II intron^26,27,47^) with well-defined high-resolution crystallographic and cryo-EM structures. Large RNA–protein complexes, such as the ribosome and spliceosome have also yielded high-resolution cryo-EM structures^26,48^. Therefore, the key outstanding issue is whether or not there are specific lncRNAs that exist with well-defined structures comparable to other large RNAs with well-defined structures. Our SAXS study of *Bvht* lays the foundational step in this direction, revealing that this RNA does possess a 3-D structure and this 3-D structure may play a role in function. Specifically, the dependence of the physical size, as estimated by *R*_g_ and *D*_max_, of the full-length *Bvht* RNA on Mg^2+^ concentration is clear evidence of the presence of tertiary contacts. In addition, we find that *Bvht* directly binds the zinc-finger protein CNBP and that the conformational ensemble of the *Bvht* RNA is significantly altered upon protein binding. The fact that our SEC-SAXS data on sub-domains of *Bvht* (e.g., 5′ and 3′ modules, MW: 30–69 kDa) did not show Mg^2+^-dependent changes in conformation (*P*(*r*) distribution shapes are similar between 6 and 12 mM Mg^2+^ in Fig. 1) suggests that Mg^2+^ may mediate inter-domain RNA–RNA tertiary interactions in the case of the full-length *Bvht* system.

Our SAXS studies of sub-regions of *Bvht*, in addition to the full-length RNA, are consistent with a modular construction of the full 3-D fold, supporting our previous 2-D chemical probing study of *Bvht*, also showing the structure to be modular. Our SAXS data are consistent with the conformational ensemble of this lncRNA containing relatively rigid, modular subsections, connected by flexible regions. We expect that our experimental strategy will be easily applicable to other lncRNAs with modular secondary structures.

To date, the only information available for the *Bvht*–CNBP complex has been that functional in vivo CNBP binding requires the 5′ asymmetric G-rich internal loop (*RHT*/*AGIL* motif) of *Bvht*^3^. As mentioned, our in vitro data suggests that for high-affinity binding, CNBP requires the full-length *Bvht*, including the 5′ module (which contains the *RHT/AGIL* motif), the central module (which contains the multiway junction), and the 3′ module. In addition, EMSA experiments suggest that fragment 1 interacts with CNBP as well (Supplementary Table 1). Interestingly, while the fragment 1 of *Bvht* alone (positions 1–358) was enough for CNBP binding (Table 4), the 5′ module of *Bvht* (positions 1–98) alone was not enough for the CNBP binding when we analyzed this interaction using SEC-SAXS. These data suggest that both the *RHT*/*AGIL* motif (positions 27–37) and other structural elements (perhaps the multiway junction in position rage 38–358) are required for CNBP binding. Specifically, CNBP binding to *Bvht* requires both the 5′ module and either the central module or 3′ module of *Bvht*. This finding, combined with the modular, but flexible nature of *Bvht* is consistent with a functional role of the conformational heterogeneity that may be required for the efficient binding of proteins.

In addition, CNBP appears to bind to full-length and fragment 1 of *Bvht* with equimolar ratio, respectively, based on the fact that we loaded *Bvht* and CNBP with equimolar ratio and in light of their *D*_max_ values (Table 4). To date, knowledge about stoichiometry between lncRNAs and their protein binding partners has been quite sparse^3,17^. We note that our estimation of the equimolar binding of *Bvht* and CNBP is suggestive rather than definitive for the following reasons. It is known that RNA and DNA scatter more strongly than proteins. Therefore, we would not be surprised even if we cannot clearly observe CNBP with this low resolution of SAXS (~13.4–38.5 Å). Furthermore, in light of previous size exclusion chromatography studies^49^, we know that RNAs tend to have much larger effective *R*_g_ than proteins do. In addition, CNBP (21.5 kDa MW) is much smaller than *Bvht* (206 kDa MW).

Regarding RNA–protein interactions, the CNBP protein is believed to function mainly inside the cell, binding to nucleic acids and controlling transcription and translation^50^. Our study provides an important stepping stone in understanding *Bvht* and CNBP function at the molecular level. While our study has helped to elucidate the *Bvht*–CNBP interaction, a more thorough investigation will be required to delineate the exact interaction points on RNA and protein, as well as the role of dynamics in the interaction. As CNBP aids in transcriptional control, future studies of lncRNA–chromatin interactions and the role of lncRNAs in chromatin looping may shed more light on CNBP function^44^. For example, it will be more informative once we learn the binding ratios between different lncRNAs and nucleosomes/chromatin. All these efforts will contribute to better decipher biological functions of noncoding RNAs.

While we find *Bvht* to have a defined 3-D structure and a highly organized secondary structure, it is also flexible. This behavior is not unlike the SAM-I riboswitch, which also has well-defined secondary and tertiary structure (in particular, a high-resolution crystal structure in the ligand-bound state), but is highly flexible in its apo state. In fact, many structured RNAs can adapt multiple conformations and are highly flexible^1,2,35^. For example, even with 300 kV cryo-electron microscopy (cryo-EM) using an energy filter, Zhang et al. could obtain only a ~9 Å resolution map of 30 kDa RNA (47 nucleotide dimer)^51^. In their report, the internal structural flexibility of the RNA limited the cryo-EM resolution and this hypothesis is supported by molecular dynamics simulation. With respect to these findings, it is not surprising that the *Bvht* RNA (206 kDa MW) would have more flexible regions than this 30 kDa RNA, as shown in our atomistic models (Supplementary Movie 2, Supplementary Fig. 6). Indeed, Zhang et al. summarized that relatively large RNAs (e.g., >200 kDa) may have flexible conformations. Even the 116 kDa MW RNA (fragment 1 of *Bvht*) shows flexible conformations (Supplementary Fig. 11). These dynamic conformations are what may confer diverse biological influences of RNAs, such as transient binding.

The flexibility of the *Bvht* lncRNA emphasizes the importance and advantage of using SAXS to investigate 3-D structures of lncRNAs. The structural information on flexible regions that are often not resolved by X-ray crystallography is apparent in SAXS-based low-resolution studies. Our study allowed us to compare representatives of individual conformational clusters to evaluate the nonuniqueness of the SAXS-based reconstruction to decipher whether there are different conformations of the lncRNA^52^. SAXS also provides estimates of shape parameters such as *R*_g_ and *D*_max_ of biological macromolecules in solution^14^ (Tables 1–4). In addition, we emphasize that SAXS-based structure data shows more physiologically relevant structures, avoiding packing effects present in crystallographic studies. In fact, it is also known that a “true solution” state (e.g., NMR) differs from even a “frozen-solution” state (cryo-EM)^51^. While the intrinsic flexibility of *Bvht* likely provides the dominant contribution to its high *R*_g_ values (for example, *Bvht* fragment 1 of 358 nt has a 78.4 Å *R*_g_), the fact that SAXS studies are performed in solution also contributes. For comparison with other RNA crystal structures of similar molecular weight, the group II intron lariat (PDB id 4R0D, 622 nts), has a *R*_g_ of only 41 Å. In a second example, 500–700 nt subregions of crystal structures of the small subunit of the ribosome (e.g., PDB id 4GKK) have *R*_g_ values between 39 and 57 Å, resulting from the tightly packed tertiary folds of ribosomal RNA. Finally, SAXS allows the use of more physiological buffers in real-time (chemical additives for crystallization and surfactant and carbon for cryo-EM grid optimization are not required), allowing the observation of structural changes resulting predominantly from certain conditions (such as Mg^2+^ concentration differences) more clearly. Similarly, we are confident that our samples were monodisperse and homogeneous (interpretation of SAXS data itself would have been extremely difficult if it were polydisperse).

Regarding secondary structure, RNA secondary structure prediction accuracy can be greatly improved by covariation-based constraints^7,9^. However, compared to protein-coding genes, long noncoding RNAs tend to have weaker sequence conservation^53^ and identifying homologs can be challenging^7^. Recently developed covariance tools^54^ reveal structure conservation in lncRNAs^55^. However, in the case of *Bvht*, no homolog has been identified to date. Our SAXS study helps to confirm the lncRNA secondary structure determined by 3S SHAPE and DMS chemical probing experiments. In particular, our ensemble of atomistic 3-D models of the *Bvht* RNA is highly consistent with the SAXS data. Since the 3-D models are based on the secondary structure, the SAXS experiments bolster the secondary structure. Our approach of using chemical probing and SAXS for modeling 3-D lncRNA structures complements a wide variety of approaches used for RNA modeling^30,31,56–59^. Indeed, it has been difficult to predict 3-D structures accurately without secondary structural constraints^60,61^.

Overall, we have shown that physiologically relevant three-dimensional SAXS-based structures of long noncoding RNAs can be determined in spite of their considerable length and flexible conformations. Our approach for this characterization is unique since it combines several biophysical/computational methodologies in an analogous fashion to Huang et al.^62^: (i) in vitro transcription of long noncoding RNA, (ii) SEC-SAXS experiments to study solution structures, (iii) computational structure determination using SAXS and secondary structure information as restraints, (iv) transformation of SAXS *DAMFILT* files into cryo-EM style maps for superposition, (v) construction of simulated solution structures from atomistic cartesian coordinates using in-house *PHENIX*^63^ scripts, and (vi) resolution estimation and flexible fitting using programs which were developed for cryo-EM map applications. Our atomistic model is also the longest isolated RNA (e.g., 636 nucleotides) to date, with the next longest RNA being 622–625 nucleotides to our knowledge^27,64^. To corroborate our findings, we performed EMSA analysis and used measured SHAPE reactivities. Our approach is broadly applicable to other RNA systems and lays the foundation for similar studies in the widely expanding classes of long noncoding RNAs, viral RNAs and mRNA–protein complexes.

## Methods

### Sample preparation

We prepared RNA samples using snapcool refolding^65^ immediately before experimental characterization (e.g., EMSA and SAXS). The full sequence of *Bvht* is in Supplementary Note 1. The CNBP coding sequence (Supplementary Note 2) was cloned into pET-28a (*MilliporeSigma*) with a C-terminal 6-histidine tag and expressed in the *ArcticExpress* (*Agilent*) strain of *E. coli*. Isopropyl β-d-1-thiogalactopyranoside (IPTG) was added at 0.4–0.8 OD_600_ to induce expression for either 3 h at 37 °C or overnight at 13 °C. Cell pellets were sonicated, and after additional centrifugation, the supernatant was applied to a Ni-NTA column (*GE Healthcare*).

### Electrophoretic mobility shift assay (EMSA)

We performed EMSA to study *Bvht*–CNBP complex migration using a 6% polyacrylamide gel containing 0.5× TBE (45 mM Tris, 45 mM Boric acid, 1 mM EDTA disodium salt, pH 8.3) and 2 mM MgCl_2_. EMSA was performed on ice at 130 V for 4 h or 100 V for 6 h. Gels were stained with ethidium bromide for 5 min and destained with water several times. Gel images were scanned with a *Hitachi FMBioII* fluorescence imager (532 nm laser excitation, 605 nm bandpass emission filter) at 100 µm resolution and −2.85 mm focus. The original image of the Fig. 1 gel is in Supplementary Fig. 12.

### SAXS experiment

In order to collect data for the monodispersed sample, devoid of any high-molecular-weight aggregates or degraded material, we performed SAXS data collection using a SEC^66^, controlled by an Agilent HPLC-SAXS set-up at the B21 beamline, Diamond Light Source (Didcot, UK). An *Agilent 1200* (*Agilent Technologies*, Stockport, UK) in-line HPLC system was connected to a specialized flow cell and an absorbance detector. 50 µL of each sample (*Bvht* at 0 mM Mg^2+^, *Bvht* at 6 mM Mg^2+^, *Bvht* at 12 mM Mg^2+^, and *Bvht* + CNBP at 6 mM Mg^2+^) was injected into a *Shodex KW403-4F* SEC column (*Showa Denko America Inc*.) which had been pre-equilibrated with sample buffer (50 mM HEPES-KOH, 100 mM KCl, pH 7.6, and either 0, 6, or 12 mM MgCl_2_). Injected sample concentrations are in Supplementary Table 2. The SEC-separated sample was exposed to X-rays, followed by data collection every 3 s.

### SAXS data processing

Using *ScÅtter*^67^, the sample peak regions that were selected were then buffer subtracted and merged using either *ScÅtter* or *Primus* in the *ATSAS* suite^68^. The *CRYSOL*, *DAMAVER*, *DAMCLUST*, *DAMMIN*, *GNOM*, *SUPCOMB*, programs in the *ATSAS* suite were used. Molecular physical properties were calculated using software modules from *ATSAS*. Guinier and Kratky analyses were performed to ensure that samples are homogenous and well-folded, respectively. The *GNOM* program was used to determine *D*_max_ and *R*_g_ by calculating the pair-distance distribution *P*(*r*) plot^29,69,70^. We estimate *D*_max_ in accordance with Trewhella et al.^39^ and note that the general decrease in *D*_max_ for full-length *Bvht* with increasing Mg^2+^ concentration is also observed with complementary methods (e.g., analytical ultracentrifugation) for other lncRNAs^5,9^ (see Supplementary Table 3 for other details). The low-resolution structures were calculated using the *DAMMIN* program^71^. For each sample, >15 low-resolution models were calculated, followed by alignment and averaging of each set of models using the program *DAMAVER* to obtain a representative shape. We also performed a clustering calculation to identify likely clusters of full-length RNA in 6 mM MgCl_2_ buffer using the *DAMCLUST* program. We converted the reconstructed bead models into electron density maps (as in cryo electron microscopy) with the program *Situs*^72^. We used a Gaussian kernel width of 6 Å and a voxel spacing of 1 Å.

### Resolution of SAXS based Solution Structure

Exact resolution estimation is difficult with typical SAXS measurements (e.g., SAXS “resolution” is ambiguous, not directly related to 2*π*/*q*). We can estimate the resolution of our SAXS based solution structures at ~13.6–37.2 Å (*DAMAVER* derived solution structures tend to have higher resolution than *DAMCLUST* ones) using *phenix.mtriage*^63^. Although the *phenix.mtriage* is being actively used for cryo-electron microscopy maps, we found that it is applicable to our SAXS based solution structure as well. For example, when we filtered the volume with a gaussian by *UCSF Chimera*^73^, it reasonably reflected decreased resolution. Although we estimate the resolution, SAXS based solution structural data should not be overinterpreted.

### Atomistic structure modeling of *Bvht*

To model atomistic RNA structures, we used a two-step procedure that mimics hierarchical folding^11^: starting from the published secondary structure of *Bvht*, we inserted a few additional base-pairs with *RNAfold*^74^. Published SHAPE and DMS data^3^ were used as soft constraints. The reason that we added more base-pairs on top of published base-pairs is that it is unlikely that long single stranded regions would not form at least some noncanonical on- and off-interactions. We then assembled known RNA fragments using a Monte Carlo algorithm to build the tertiary structure. The idea of fragment assembly is well established^75^ and has been used to predict models matching SAXS data by Dzananovic et al.^29^. In *ERNWIN*, we use fragments for secondary structure elements (hairpins, interior loops, multiloop segments), extracted from the representative set of RNA containing PDB structures^76^ with the help of our Python RNA structure library *Forgi*^77^. After every sampling step, we used the correlation between the pair-wise distance distribution function of the proposed tertiary structure and the experimental SAXS distance distribution function, derived with *GNOM*. To save computation time, the pair-wise distance distribution of our models was calculated using only one point per nucleotide. After sampling was complete, we used the established tool *CRYSOL* to further filter the predicted structures for the top-ranked *χ* value. In contrast to our estimated pair-wise distance distribution function used during sampling, this program takes all atoms and the hydration layer into account. To save computation time, we only evaluated every 1000th structure using *CRYSOL*. The best presented atomistic models for full-length *Bvht* at 12 mM Mg^2+^ have a *χ* value better (lower) than 1.75. We also carried out the same sampling procedure for an alternative secondary structure and for a null hypothesis system, random secondary structures (predicted from di-nucleotide shuffled sequences at higher temperatures, to roughly match the number of base-pairs in the *Bvht* secondary structure), as a control. Control structures have poorer *χ* values (Supplementary Fig. 5).

### Alignment and visualization details

We used *SUPCOMB* to align SAXS-based solution structures and models to improve chirality correctness. For Fig. 5c, d, we superimposed all 30 atomistic models with equal weight using *UCSF Chimera* (e.g., File −> Save PDB −> Save multiple models in a single file) saving an NMR style pdb file that uses a model number. When we aligned superimposed models to averaged solution structures, we used “*Fit in map*” of *UCSF Chimera*^73^. All solution structures, atomistic models, and movies were visualized by *UCSF Chimera*.

### Reporting summary

Further information on research design is available in the Nature Research Reporting Summary linked to this article.

## Supplementary information

Supplementary Information

Peer Review

Reporting Summary

Description of Additional Supplementary Files

Supplementary Movie 1

Supplementary Movie 2

Supplementary Movie 3

## Data Availability

All of our SAXS data used for modeling have been deposited to the small angle scattering biological data bank (https://www.sasbdb.org/project/939/x6kirb9f97/)^78^. All other data including atomistic structures and cloning constructs will be made available from the corresponding author upon reasonable request.

## Code availability

All codes (e.g., *ERNWIN* and in-house python codes for SAXS data processing) are available at https://github.com/pkerpedjiev/ernwin and https://github.com/kimdn/in_house_codes_for_SAXS.
