# Supplementary material for: Zinc-finger protein CNBP alters the 3-D structure of lncRNA Braveheart in solution
Source: Nat Commun. 2020 Jan 9;11:148. doi: 10.1038/s41467-019-13942-4 (PMC6952434; doi:10.1038/s41467-019-13942-4)
Supplement: Supplementary file 1 — Supplementary Information [file 41467_2019_13942_MOESM1_ESM.pdf]

Supplementary Information

Zinc-finger protein CNBP alters the 3-D structure of lncRNA *Braveheart* in solution

Kim et al.

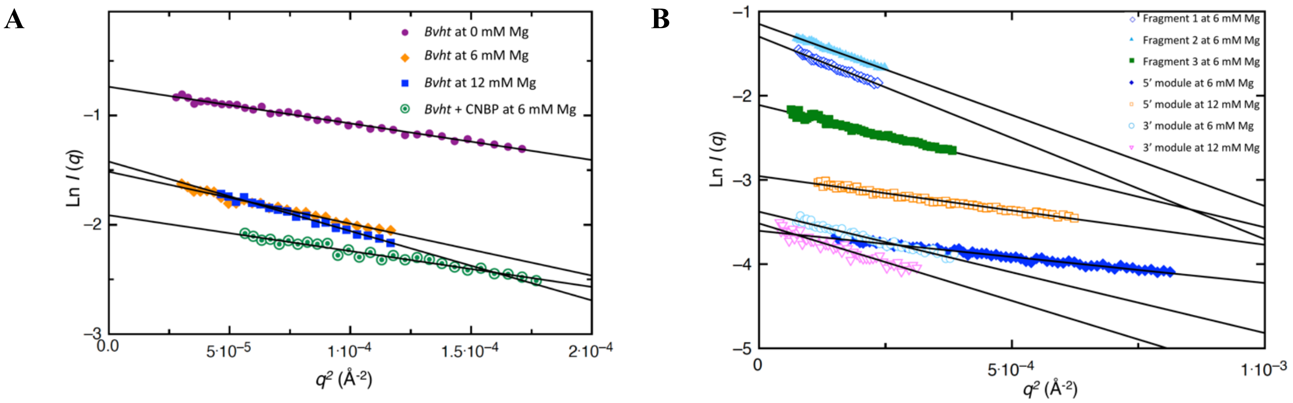

Supplementary Figure 1. Guinier analysis of *Bvht.* (A) Full-length *Bvht* at various magnesium concentrations and also *Bvht*-CNBP complex at $[Mg^{2+}]$ = 6 mM. (B) *Bvht* sub-regions. The plots are approximately linear in the Guinier regime, a sound indicator of mono-dispersity (non-aggregation).

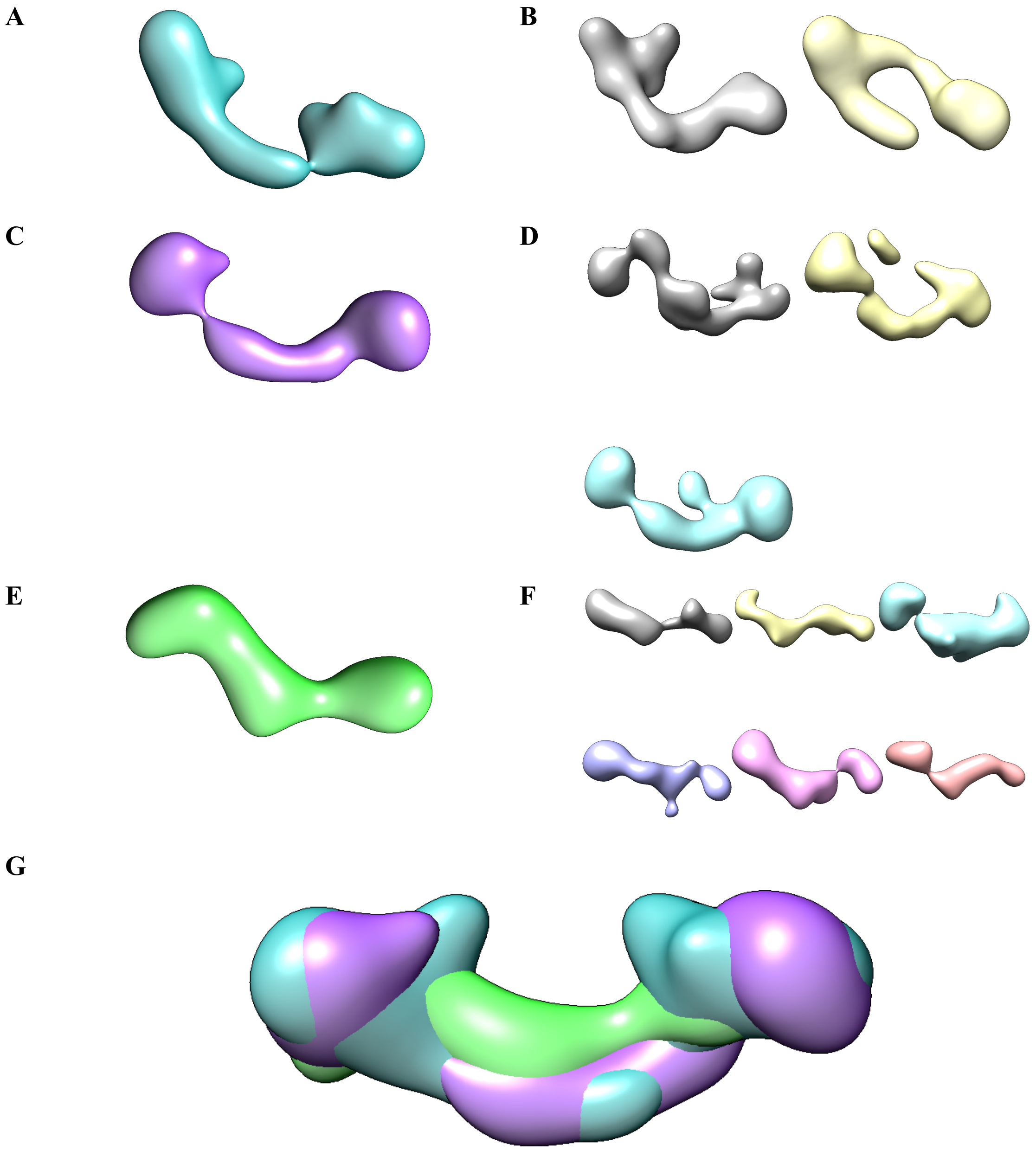

Supplementary Figure 2. Solution structures of full-length *Bvht* at various Mg^{2+} concentrations. (A) Averaged structures acquired by *DAMAVER* at 0 mM Mg^{2+}. (B) Each class of structures obtained by *DAMCLUST* at 0 mM Mg^{2+}. (C) Averaged structures acquired by *DAMAVER* at 6 mM Mg^{2+}. (D) Each class of structures obtained by *DAMCLUST* at 6 mM Mg^{2+}. (E) Averaged structures acquired by *DAMAVER* at 12 mM Mg^{2+}. (F) Each class of structures obtained by *DAMCLUST* at 12 mM Mg^{2+}. (G) Superposition of 0 mM Mg^{2+}, 6 mM Mg^{2+} and 12 mM Mg^{2+} averaged conformations.

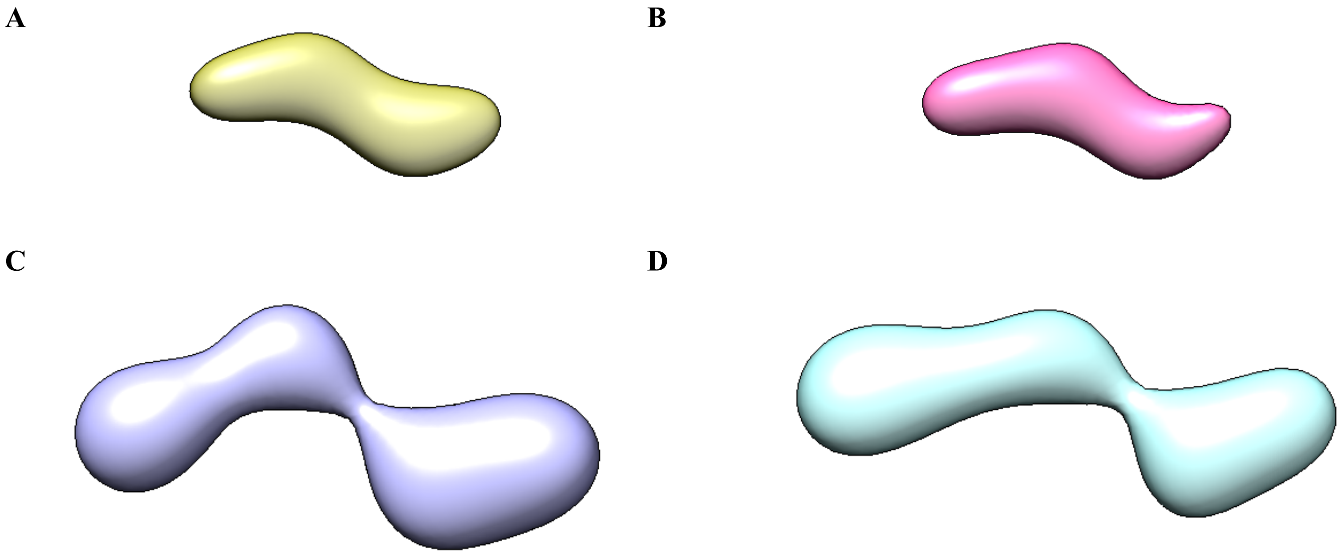

Supplementary Figure 3. Averaged structures of *Bvht* modules. (A) 5' module of *Bvht* at 6 mM Mg^{2+} acquired by *DAMAVER*. (B) 5' module of *Bvht* at 12 mM Mg^{2+}. (C) 3' module of *Bvht* at 6 mM Mg^{2+}. (D) 3' module of *Bvht* at 12 mM Mg^{2+}.

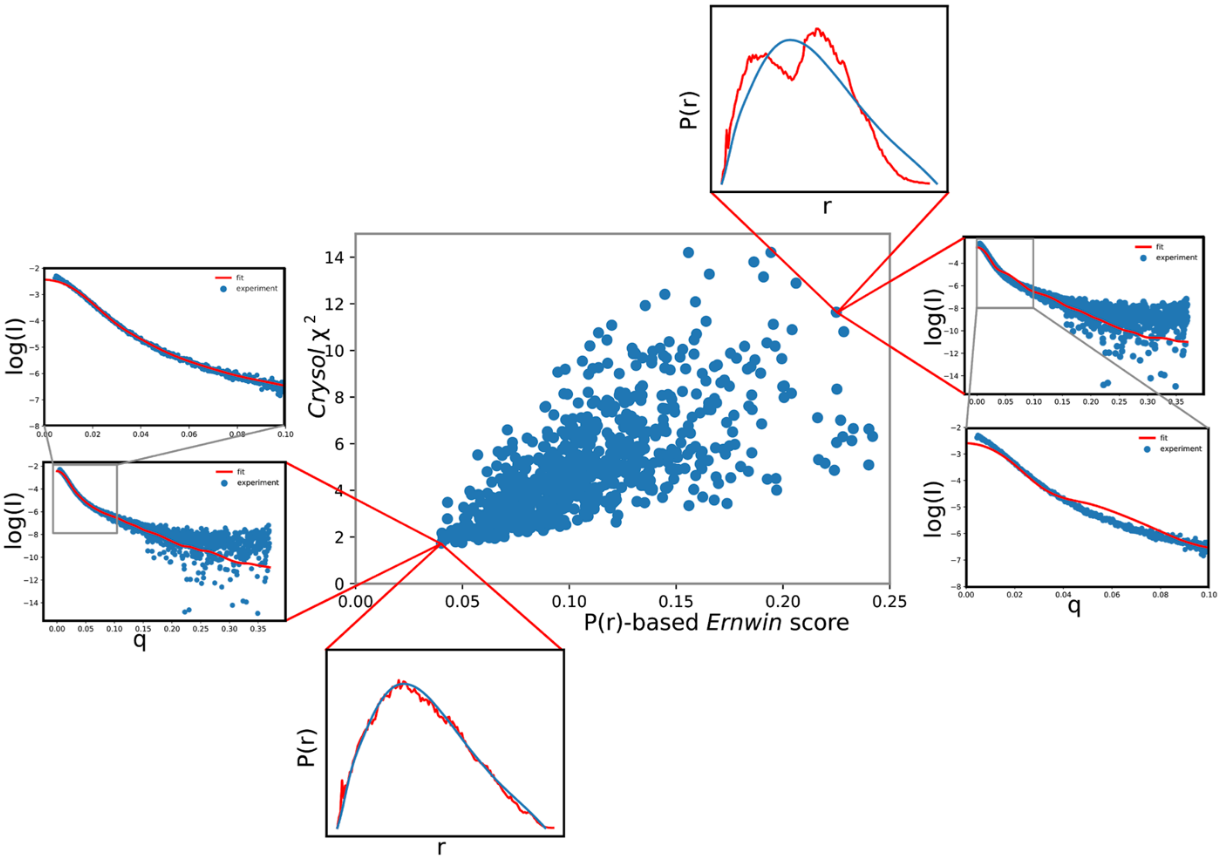

Supplementary Figure 4. Refinement of SAXS-based low-resolution structures using computation. Best-fit model in lower-left corner. The central plot shows the **χ** value calculated by *CRYSOL* on the y-axis and the *P(r)*-based *ERNWIN* score on the x-axis. The *P(r) ERNWIN* score is based on the coarse-grained (1 bead per nucleotide) pair-distance distribution used during sampling in *ERNWIN*. In theory, there should be a 1:1 correspondence between the *P*(*r*) and *I*(*q*) plots. However, in *ERNWIN* we used only one bead per nucleotide (to speed up computation) and neglect the effect of the hydration shell. This plot shows that, despite these simplifications, optimizing the *P*(*r*) score is sufficient to improve the all-atom **χ** values (which include the effect of the hydration shell). Each dot in the central plot corresponds to one predicted tertiary structure constrained to the experimentally determined secondary structure. Tertiary structures were taken from all points of the trajectory, not only after convergence. The lower left outer three plots depict the scattering intensity (*log(I(q))*) and pair-distance distribution (*P(r)*) for the best fit. The upper right outer three plots depict the scattering intensity and pair-distance distribution for one of the poorest fits. For the pair-distance distributions, the experimental *P*(*r*) curve (from *GNOM*) is shown in blue and the coarse-grained *ERNWIN*-plot of the predicted structure is shown in red. The *P(r)*-based *ERNWIN* score is difference in areas underneath the blue and red curves. At the left and right, the fit of *I(q)* for the predicted model (red) to the experimental scattering data (blue) calculated by *CRYSOL* is shown, including a zoom-in to the most relevant region at smaller angles. Simulated *P*(*r*) distributions (red) are calculated using 3-D coarse-grained models (one bead per nucleotide) consistent with SHAPE-determined *Bvht* secondary structures. 3-D motif-based homology modeling is used to produce atomistic models from coarse-grained models. **χ**-values are calculated by *CRYSOL* using all-atom structures. The previously published secondary structure was used with a few additional base-pairs introduced from *RNAfold* (taking into account the SHAPE reactivity).

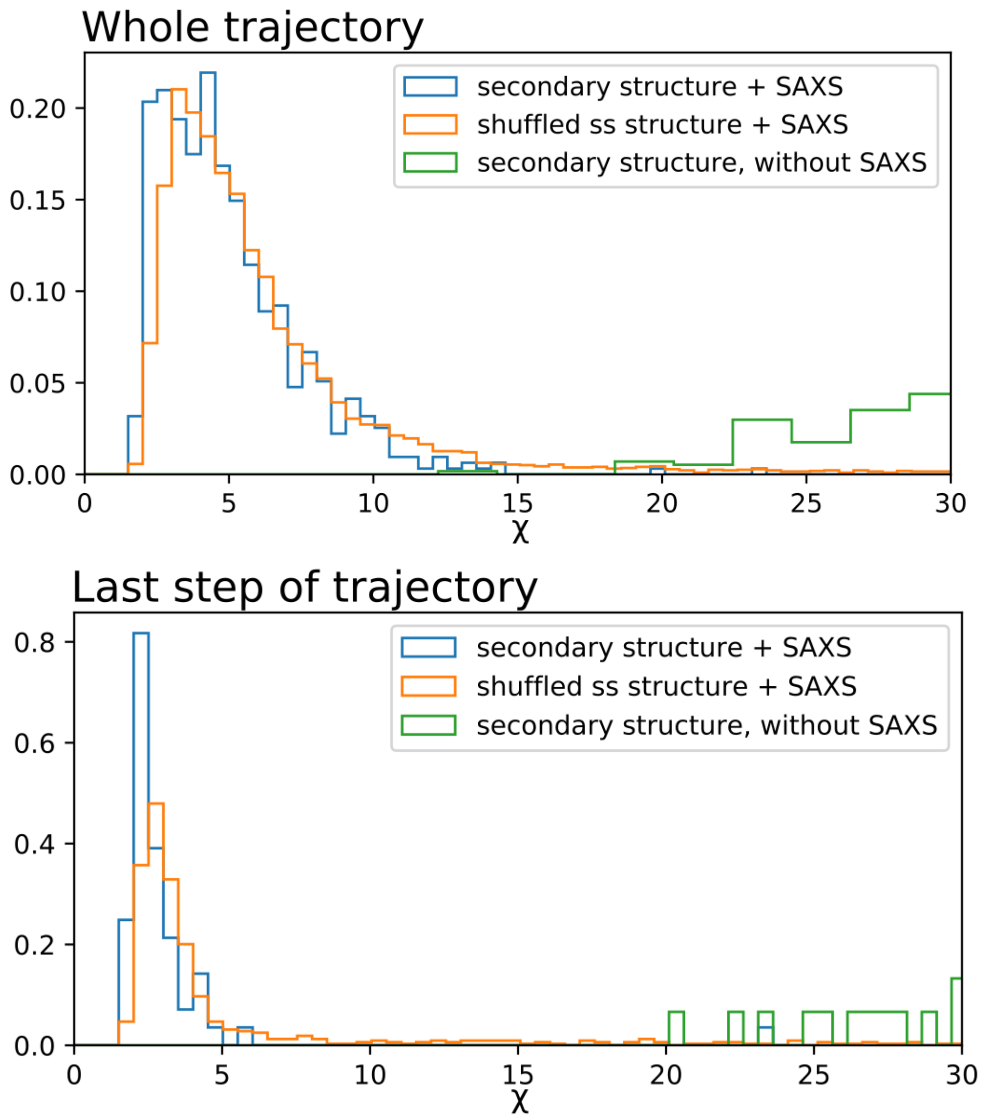

Supplementary Figure 5. χ of SAXS-based 3-D structures prefers published 2-D structure over null hypothesis. **χ** values (represented as histograms) are calculated by *CRYSOL*. Previously published secondary structure is based on chemical probing experiments. Null hypothesis is based on shuffled secondary structure. Top panel: histograms of structures from the whole trajectory for multiple trajectories. Blue, *ERNWIN* samples using the published secondary structure plus a few additional base-pairs predicted by *RNAfold* (taking into account SHAPE reactivity) and SAXS data as restraints. Orange: *ERNWIN* samples using random secondary structures plus SAXS data. Green: Sampling with the same secondary structure as for blue, but without SAXS data. Bottom panel: structures from the end of the trajectory, when sampling has converged. It can be seen that the presumably correct secondary structure leads to slightly better *CRYSOL*-**χ** values than random secondary structures, especially after sampling has converged. However, due to the large number of degrees of freedom, it is possible to find some well-fitting conformations even for some random secondary structures.

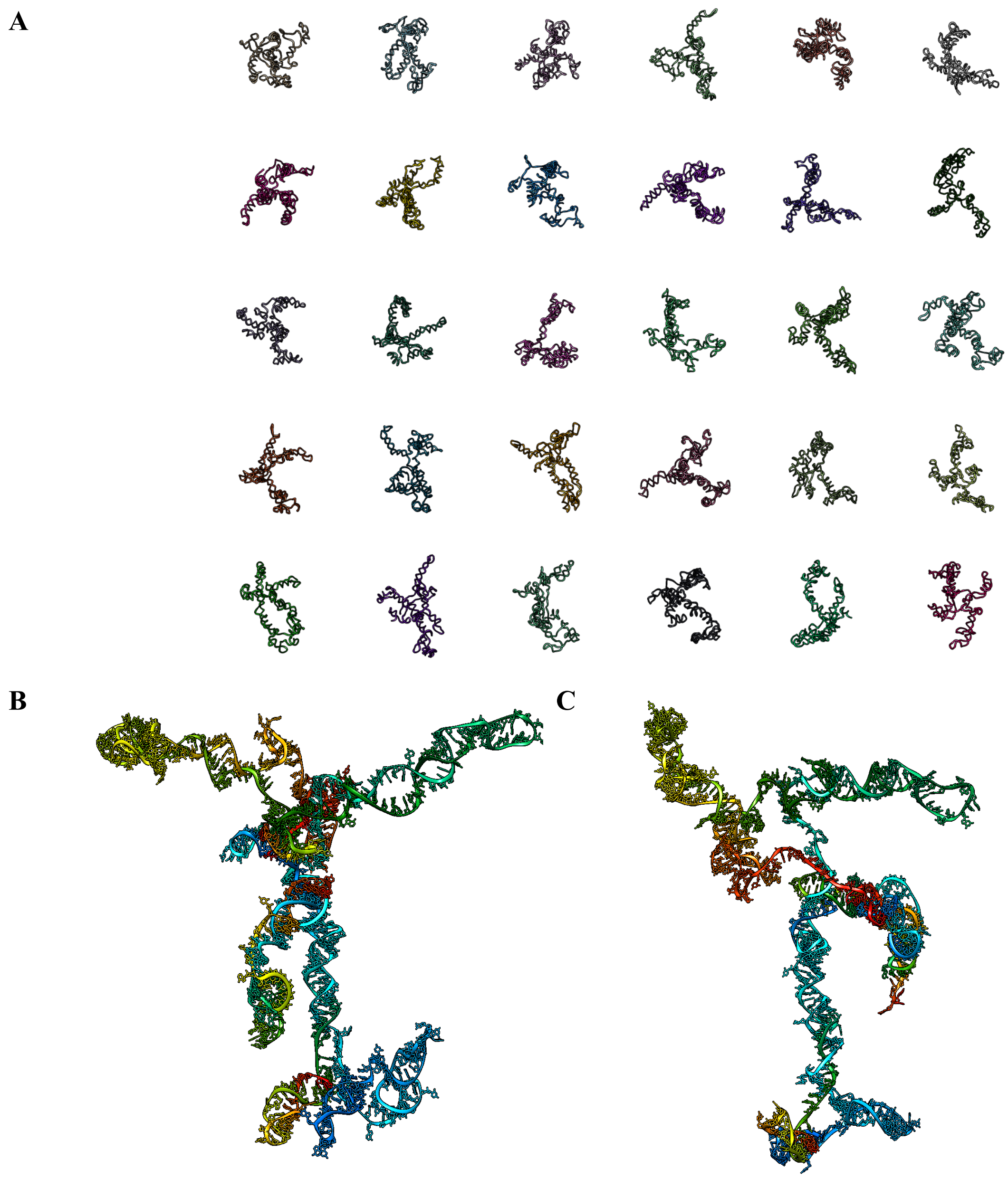

Supplementary Figure 6. Atomistic models of full-length *Bvht* at 12 mM Mg^{2+}. A) 30 top-ranked atomistic models. B) Representative structure (16 members), after clustering. Clustering was performed with *UCSF Chimera*. C) A second representative structure (16 members as well), after clustering.

A 0º 180º

B 0º 180º

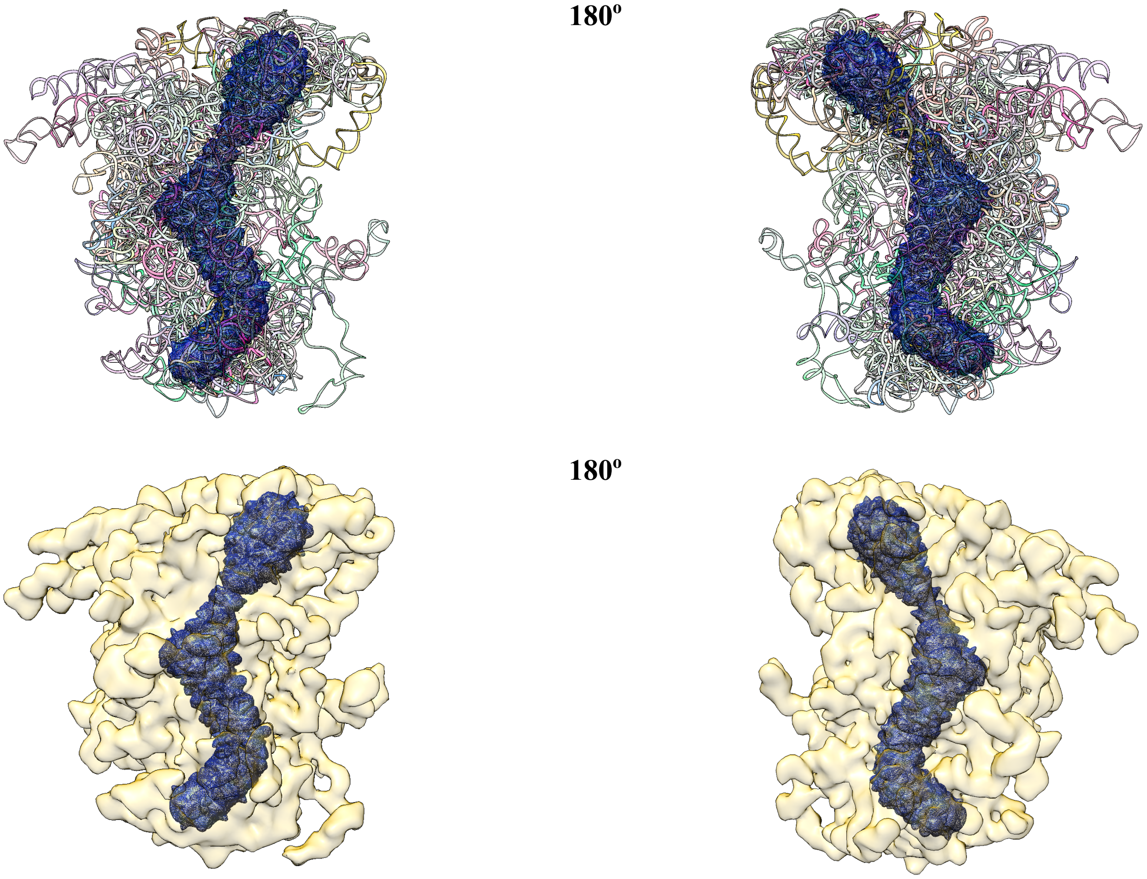

Supplementary Figure 7. Superimposed top 30 **χ** atomistic models of full-length *Bvht* at 12 mM Mg^{2+} (are in Supplementary Fig. 6). (A) Top 30 **χ**-ranked atomistic models ($1.7 < \chi < 2.6$) in various semi-transparent colors. Blue mesh, SAXS experiments (*DAMAVER* averaged structure of full-length *Bvht* at 12 mM Mg^{2+}). (B) Simulated solution structure of these top 30 models, at a contour level showing density for all atoms in the RNAs (yellow). The atomistic models are consistent with a highly flexible *Bvht*. At a higher contour level (*e.g.* contour = 6.45), corresponding the most densely populated region, close agreement between the atomistic model ensemble and experimentally derived SAXS-based 3-D map is obtained (Fig. 5).

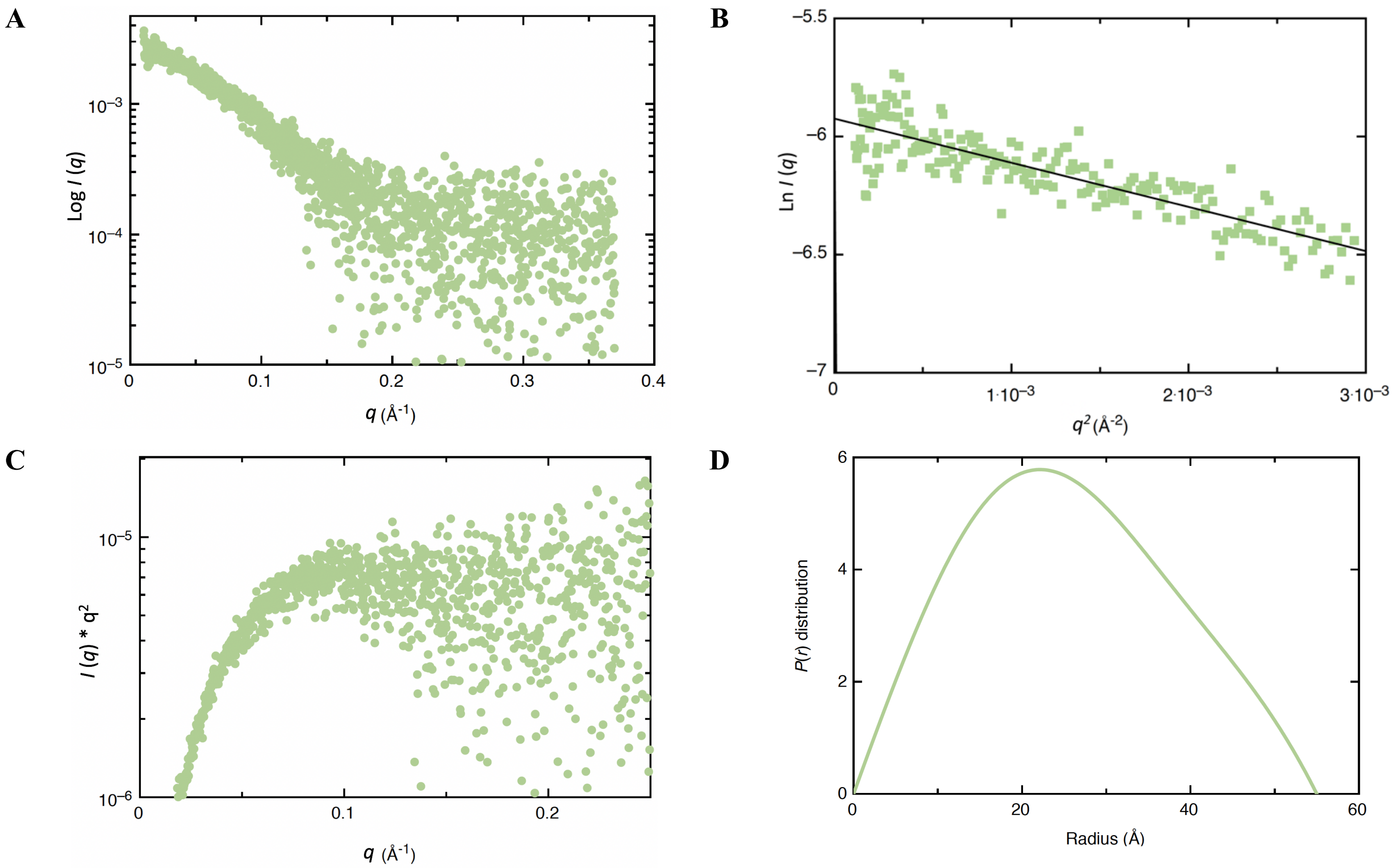

Supplementary Figure 8. Small angle X-ray scattering data for CNBP only. (A) Scattering intensity versus scattering angle ($q = 4\pi\sin\theta/\lambda$). (B) Guinier plot depicting ln (I(q)) vs. q^2. (C) Kratky plot suggests that CNBP is not a disordered protein. (D) Pair-distance distribution function shows an overall globular structure of CNBP.

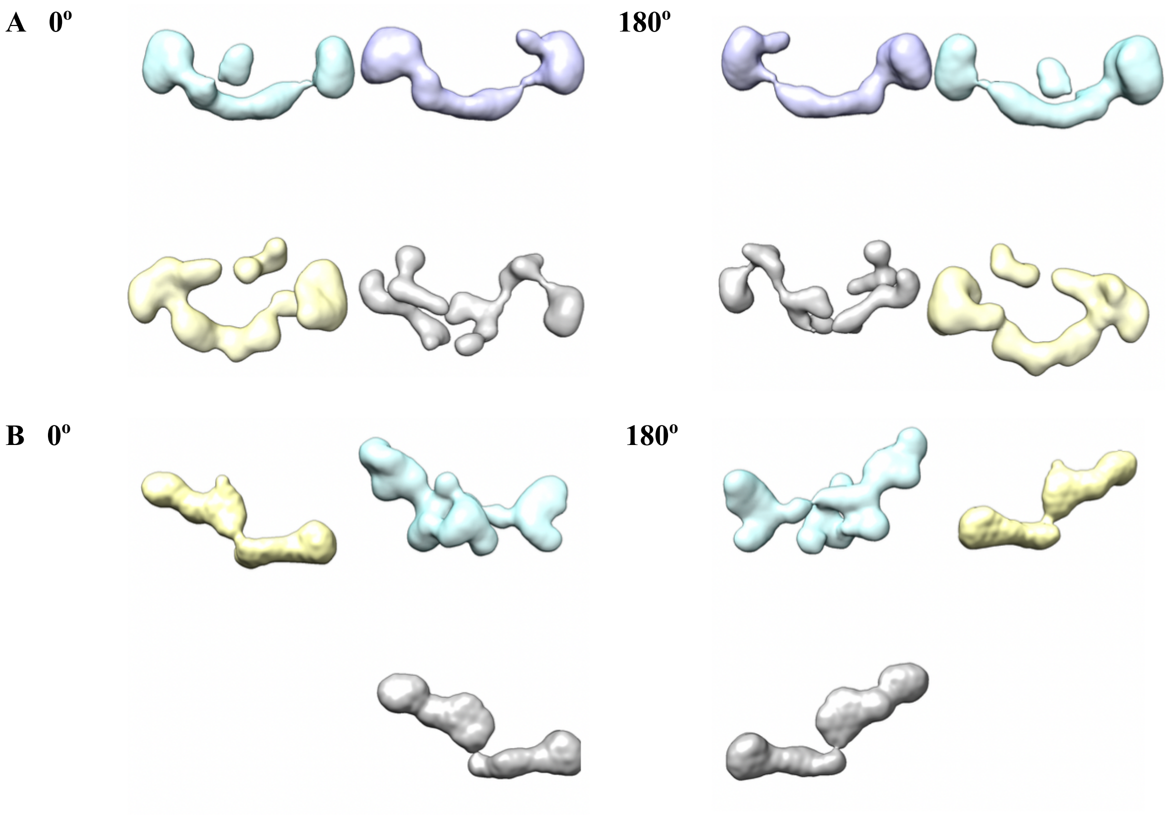

Supplementary Figure 9. Effect of CNBP binding on conformation of full-length *Bvht* at 6 mM Mg^{2+}. (A) full-length *Bvht*. (B) Full-length *Bvht*-CNBP complex.

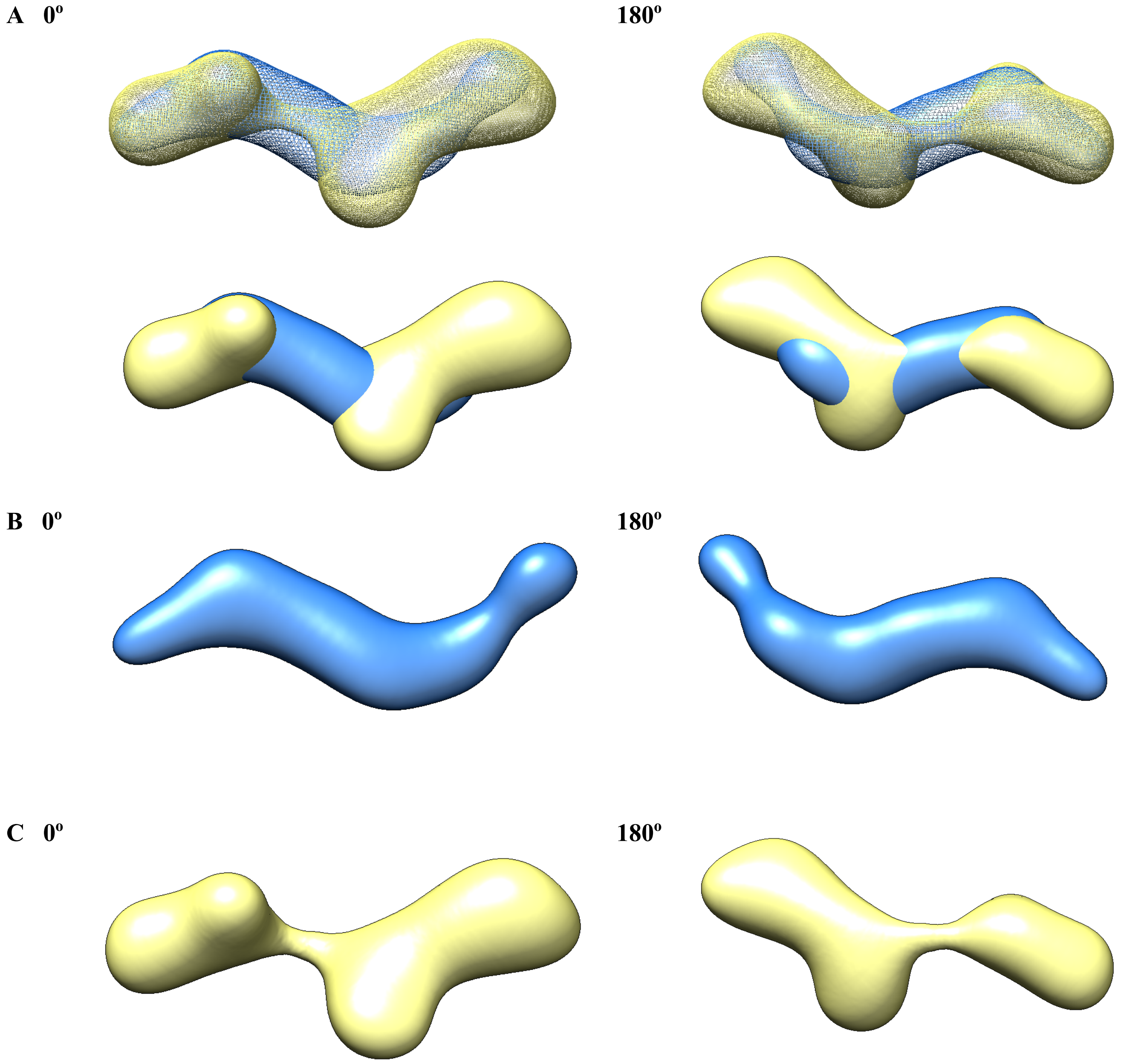

Supplementary Figure 10. Solution structures of complex of CNBP with fragment 1 of *Bvht*. (A) Blue, averaged solution structure of fragment 1 of *Bvht* only; yellow, averaged solution structure of fragment 1 of *Bvht*-CNBP complex. (B) Fragment 1 of *Bvht* only. (C) Complex of CNBP with fragment 1 of *Bvht*. Individual solution structures of fragment 1 of *Bvht* only are presented in Supplementary Fig. 11.

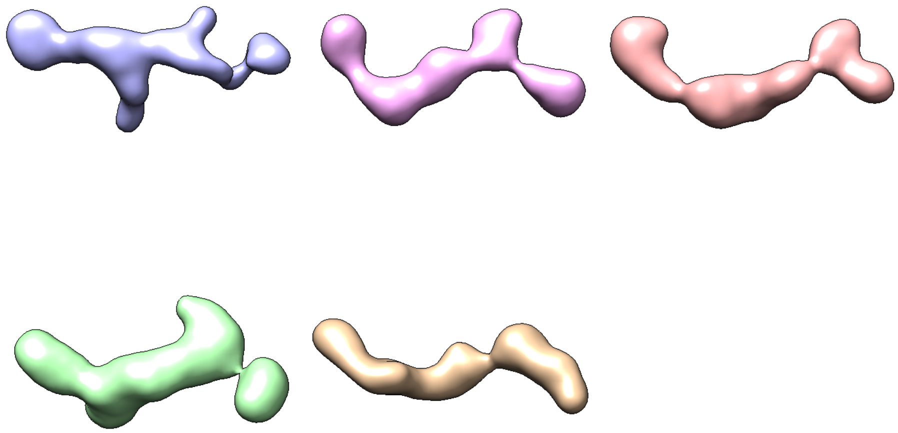

Supplementary Figure 11. Solution structures (not averaged) of fragment 1 of *Bvht* at 6 mM Mg^{2+}. These structures were obtained by *DAMCLUST*.

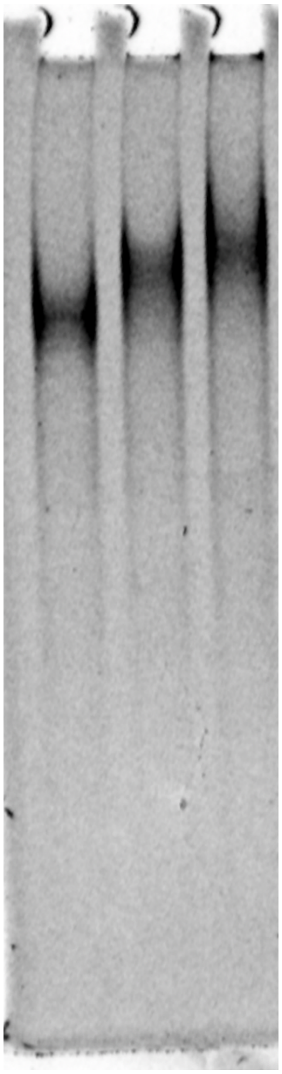

Supplementary Figure 12. The original EMSA gel picture of Fig. 1.

Bvht	**Fragment 1**					**Fragment 2**			**Fragment 3**		
Lane #	1	2	3	4	5	6	7	8	9	10	11
Bvht [μM]	0.20	0.20	0.19	0.18	0.16	0.05	0.05	0.05	0.05	0.05	0.05
CNBP [μM]	0.00	0.50	1.00	2.00	4.00	0.00	0.84	1.67	0.00	0.84	1.67

EMSA image

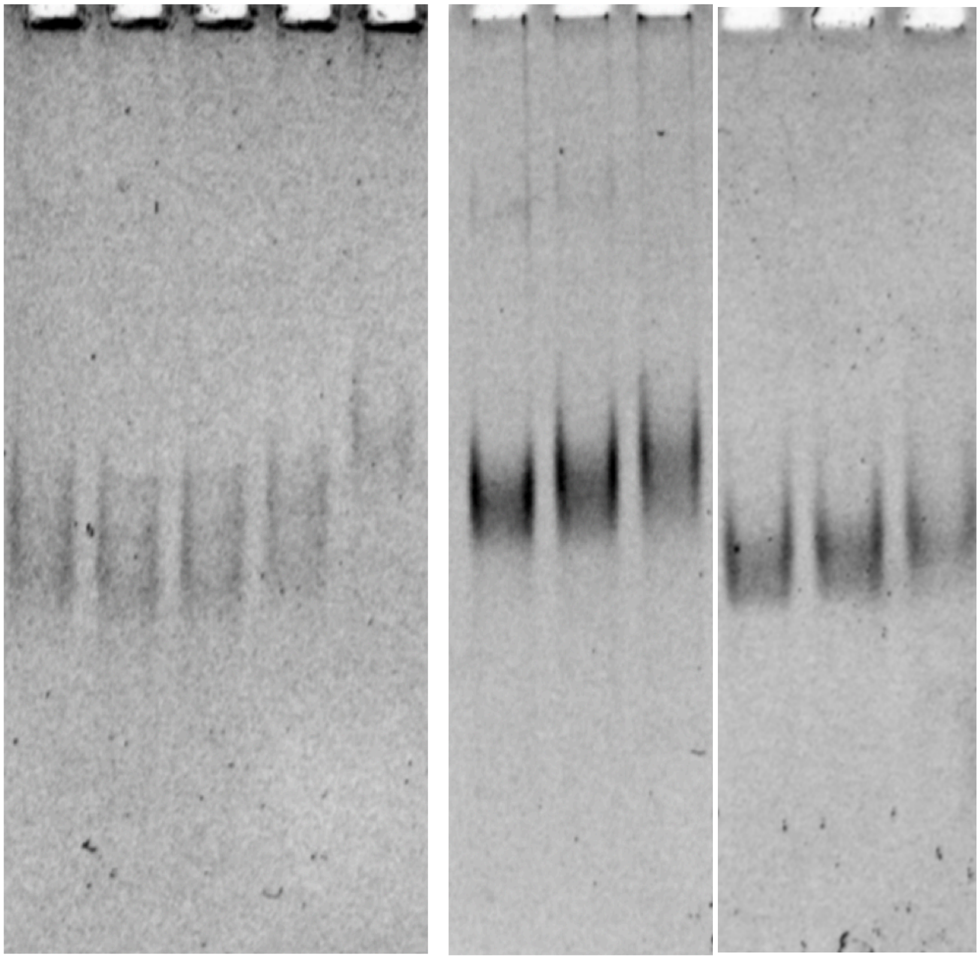

Supplementary Table 1. EMSA to evaluate binding of *Bvht* fragments with CNBP. EMSA stands for electrophoretic mobility shift assay. The shift for fragments of *Bvht* upon addition of CNBP is less pronounced in comparison to EMSA studies of full-length *Bvht*.

Sample	Mg^{2+} Concentration	Sample Concentration
Full-length *Bvht*	0 mM	1.90 mg/ml
	6 mM	2.00 mg/ml
	12 mM	1.60 mg/ml
Full-length *Bvht* + CNBP	6 mM	0.86 mg/ml
5' module of *Bvht*	6 mM	1.87 mg/ml
	12 mM	1.42 mg/ml
Central module of *Bvht*	6 mM	N/A
	12 mM	1.12 mg/ml
3' module of *Bvht*	6 mM	1.39 mg/ml
	12 mM	0.97 mg/ml
Fragment 1 of *Bvht*	6 mM	1.87 mg/ml
Fragment 2 of *Bvht*	6 mM	1.16 mg/ml
Fragment 3 of *Bvht*	6 mM	0.50 mg/ml
CNBP	6 mM	2.50 mg/ml

Supplementary Table 2. Sample concentration for SEC-SAXS data collection.

Sample	Mg^{2+} Concentration	D_{max}	q_{min}	π/D_{max}
Full-length *Bvht*	0 mM	300 Å	0.0099 $Å^{-1}$	0.0104 $Å^{-1}$
	6 mM	287 Å	0.0097 $Å^{-1}$	0.0109 $Å^{-1}$
	12 mM	260 Å	0.0117 $Å^{-1}$	0.0121 $Å^{-1}$
Full-length *Bvht* + CNBP	6 mM	301 Å	0.0086 $Å^{-1}$	0.0104 $Å^{-1}$
5' module of *Bvht*	6 mM	135 Å	0.0126 $Å^{-1}$	0.0233 $Å^{-1}$
	12 mM	150 Å	0.0141 $Å^{-1}$	0.0209 $Å^{-1}$
3' module of *Bvht*	6 mM	185 Å	0.0108 $Å^{-1}$	0.0170 $Å^{-1}$
	12 mM	190 Å	0.0108 $Å^{-1}$	0.0165 $Å^{-1}$
Fragment 1 of *Bvht*	6 mM	240 Å	0.0130 $Å^{-1}$	0.0131 $Å^{-1}$
Fragment 1 of *Bvht* + CNBP	6 mM	270 Å	0.0075 $Å^{-1}$	0.0116 $Å^{-1}$
Fragment 2 of *Bvht*	6 mM	271 Å	0.0099 $Å^{-1}$	0.0116 $Å^{-1}$
Fragment 3 of *Bvht*	6 mM	205 Å	0.0119 $Å^{-1}$	0.0153 $Å^{-1}$

Supplementary Table 3. Validation of D_{max} determination. D_{max} was estimated to be the value of r when the corresponding *P*(r) approaches zero. Our minimum *q* values (scattering vector) measured from SAXS experiments are less than π/D_{max} in all cases, consistent with the requirement discussed in Trewhella et al. that, for a particle with maximum dimension D_{max}, the minimum *q* value measured should be at most $\sim\pi/D_{max}$.

Supplementary Note 1. Sequence of *Bvht* used for SHAPE, DMS probing and SAXS (636 nt). The last 46 nucleotides in green background are not part of *Bvht*. These were added as 3' structure cassette for primer extension after SHAPE and DMS probing.

5’-
GGAUCUCUGCCCCUCAGAGUCCUGGGGAGGAUGGAUGGAACAGGAGGAGCAUCAUGAGA
AGGCUUCCUGUAAACUACGGAGGGUGAGGGGACGGAGCGUGAGGGAACGUCGUGAGAUG
GUGCUCGUGGAAGCCAGCAGAGGGUGUAGGGUCUCCUGGAGCCACAUCUCUGGAGUUAC
AUGAAGUUGUGGACCUCUCAACAUGGGUUCUGGGAACUGAACCCAGGUCCUCUGGAAGG
ACAUCGAGUGCUCUUAACCACUGAGCCAUCUCUGCAGCCUAACCCCAUCCCUUGGAGGCU
GAAGCAAAGCAAGUUCAAUGACCGUGCGACCUGGCUCUUGUUUGGGCCUAAGGAAAAGC
CGACUGUAAGCCGGGAAGCAUCUCUGGCAAGAACAAGGAAGGAUUAAGCACGUCUGUGG
UUGUGCGCAGGGAUCACAAGGUGCUCCUGCAGUGGGAAGAGCCACCACUAGGGGCGCUCC
AAGUCUUGUAUUUUCUACCUUGAAAACUCGUUUCCCUGUGGCUGCCCUCCAAAUCCAACG
GAGAUGAAUGGCUUUUAGUUUACCUAGCUGAUUUAAUAAACUUUAAAAAUAAAUGUCCG
AUCCGCUUCGGCGGAUCCAAAUUGGGCUUCGGUUCGGUUCGAU-3’

Supplementary Note 2. Sequence of CNBP protein (177 AA) used for SAXS. The n-terminal 16 amino acids in green background are not part of CNBP protein. These were added as a N-terminal histidine tag.

N-term-
LTLRRRYTMGHHHHHHMSSNECFKCGRSGHWARECPTGGGRGRGMRSRGRGGFTSDRGFQFV
SSSLPDICYRCGESGHLAKDCDLQEDACYNCGRGGHIAKDCKEPKREREQCCYNCGKPGHLARD
CDHADEQKCYSCGEFGHIQKDCTKVKCYRCGETGHVAINCSKTSEVNCYRCGESGHLARECTIE
ATA-C-term
