# Supplementary material for: Zinc-finger protein CNBP alters the 3-D structure of lncRNA Braveheart in solution
Source: Nat Commun. 2020 Jan 9;11:148. doi: 10.1038/s41467-019-13942-4 (PMC6952434; doi:10.1038/s41467-019-13942-4)
Supplement: Supplementary file 3 — Reporting Summary [file 41467_2019_13942_MOESM3_ESM.pdf]

nature research

Corresponding author(s): Karissa Sanbonmatsu

Last updated by author(s): Aug 22, 2019

Reporting Summary

Nature Research wishes to improve the reproducibility of the work that we publish. This form provides structure for consistency and transparency in reporting. For further information on Nature Research policies, see Authors & Referees and the Editorial Policy Checklist.

Statistics

For all statistical analyses, confirm that the following items are present in the figure legend, table legend, main text, or Methods section.

n/a	Confirmed	
☐	☒	The exact sample size (n) for each experimental group/condition, given as a discrete number and unit of measurement
☐	☒	A statement on whether measurements were taken from distinct samples or whether the same sample was measured repeatedly
☐	☒	The statistical test(s) used AND whether they are one- or two-sided<br>*Only common tests should be described solely by name; describe more complex techniques in the Methods section.*
☒	☐	A description of all covariates tested
☐	☒	A description of any assumptions or corrections, such as tests of normality and adjustment for multiple comparisons
☐	☒	A full description of the statistical parameters including central tendency (e.g. means) or other basic estimates (e.g. regression coefficient) AND variation (e.g. standard deviation) or associated estimates of uncertainty (e.g. confidence intervals)
☐	☒	For null hypothesis testing, the test statistic (e.g. F, t, r) with confidence intervals, effect sizes, degrees of freedom and P value noted<br>*Give P values as exact values whenever suitable.*
☒	☐	For Bayesian analysis, information on the choice of priors and Markov chain Monte Carlo settings
☒	☐	For hierarchical and complex designs, identification of the appropriate level for tests and full reporting of outcomes
☒	☐	Estimates of effect sizes (e.g. Cohen's d, Pearson's r), indicating how they were calculated

Our web collection on statistics for biologists contains articles on many of the points above.

Software and code

Policy information about availability of computer code

Data collection	Hitachi FMBioII fluorescence imager software was used to collect EMSA images. Agilent 1200 (Agilent Technologies, Stockport, UK) in-line HPLC system software was used for SEC-SAXS data.
Data analysis	We used the latest ATSAS suite that has ScÅtter, Primus, GNOM, DAMMIN, CORAL, CRYSOL, SUPCOMB, DAMAVER programs. These programs were used to process and analyze SAXS data. We used latest version of ERNWIN and RNAfold programs to model lncRNA atomistic structures. Latest version of SWISS-MODEL, ROSETTA, Coral programs and QUARK web server (Zhang lab, Univ. Michigan) were used to model CNBP protein structure. UCSF Chimera (1.14) was used for model illlustration.

For manuscripts utilizing custom algorithms or software that are central to the research but not yet described in published literature, software must be made available to editors/reviewers. We strongly encourage code deposition in a community repository (e.g. GitHub). See the Nature Research guidelines for submitting code & software for further information.

Data

Policy information about availability of data

All manuscripts must include a data availability statement. This statement should provide the following information, where applicable:

- Accession codes, unique identifiers, or web links for publicly available datasets
- A list of figures that have associated raw data
- A description of any restrictions on data availability

All data including SAXS maps and atomistic structures and cloning constructs used in this study will be made available from the corresponding author upon reasonable request.

Field-specific reporting

Please select the one below that is the best fit for your research. If you are not sure, read the appropriate sections before making your selection.

☒ Life sciences ☐ Behavioural & social sciences ☐ Ecological, evolutionary & environmental sciences

For a reference copy of the document with all sections, see nature.com/documents/nr-reporting-summary-flat.pdf

Life sciences study design

All studies must disclose on these points even when the disclosure is negative.

Sample size	For all structural biology studies (including X-ray crystallography, NMR, Cryo-EM), it is common to use one plasmid (cloning construct) after confirming sequence. As in our previous publication (e.g. Nucleic Acids Res. 39, 2416–2431 (2011)), we prepared RNA samples. All RNA constructs and protein samples were prepared from plasmids and we confirmed their sequences with the Genewiz company. For all-atom models, several hundred models were considered, with the top 30 chosen for the final volume maps.
Data exclusions	We performed four SAXS studies, each at the same facility (DIAMOND, U.K.). The first study was a preliminary study that gave us confidence to pursue the target (bvht lncRNA). During the second study, we obtained high quality data that we used in our publication and for atomistic modelling. During the third study, we also obtained high quality data; however, we did not use all of the data from this study because data from the second study were sufficient to complete the all-atom models (performed by University of Vienna). We confirmed that the second and third studies displayed similar solution structures for the bvht lncRNA. During the fourth study, we obtained data for fragments of bvht lncRNA.
Replication	As we previously described above (Data exclusions), the 2nd and 3rd SAXS studies showed similar solution structures for the structures of bvht. Therefore, we are more confident that we reproduced our results. For all-atom models, several hundred models were produced with the top 30 chosen for the final volume maps.
Randomization	In SAXS experiments, ensemble averages are computed from a large distribution of randomly oriented molecule. Each RNA and protein molecule in solution are totally randomized with respect to its orientation. For the all-atom models, in addition to the models based on the SHAPE probing based bvht secondary structure, a null hypothesis case was considered with randomly shuffled secondary structures.
Blinding	We are not dealing with clinical trials for new drugs. Therefore, there is no reason that we need to do blind studies. Rather, proper labeling of each sample and data/file was implemented to avoid misclassification.

Reporting for specific materials, systems and methods

We require information from authors about some types of materials, experimental systems and methods used in many studies. Here, indicate whether each material, system or method listed is relevant to your study. If you are not sure if a list item applies to your research, read the appropriate section before selecting a response.

Materials & experimental systems

n/a	Involved in the study	
☒	☐	Antibodies
☒	☐	Eukaryotic cell lines
☒	☐	Palaeontology
☒	☐	Animals and other organisms
☒	☐	Human research participants
☒	☐	Clinical data

Methods

n/a	Involved in the study	
☒	☐	ChIP-seq
☒	☐	Flow cytometry
☒	☐	MRI-based neuroimaging
