# Supplementary material for: Zinc-finger protein CNBP alters the 3-D structure of lncRNA Braveheart in solution
Source: Nat Commun. 2020 Jan 9;11:148. doi: 10.1038/s41467-019-13942-4 (PMC6952434; doi:10.1038/s41467-019-13942-4)
Supplement: Supplementary file 4 — Description of Additional Supplementary Files [file 41467_2019_13942_MOESM4_ESM.docx]

**Description of Additional Supplementary Files**

File name: Supplementary Movie 1
Description: Superposition of atomistic models of full-length Bvht at 12 mM Mg2+.

File name: Supplementary Movie 2
Description: Three top-ranked atomistic structures show flexibility even after alignment.

File name: Supplementary Movie 3
Description: Superposition of atomistic models of Bvht 5' module at 6 mM Mg2+.
