# Supplementary material for: Zinc-finger protein CNBP alters the 3-D structure of lncRNA Braveheart in solution
Source: Nat Commun. 2020 Jan 9;11:148. doi: 10.1038/s41467-019-13942-4 (PMC6952434; doi:10.1038/s41467-019-13942-4)
Supplement: Supplementary file 2 — Peer Review [file 41467_2019_13942_MOESM2_ESM.pdf]

Reviewers' comments:

Reviewer #1 (Remarks to the Author):

Determination of the tertiary structure of an RNA remains a challenging experimental and computational problem that has no simple solution. Here, the authors attempt to describe the folded structure(s) of a large noncoding RNA, Braveheart (Bvht), from SAXS data and modeling. There is no atomistic structure of Bvht in its tertiary fold, although a secondary structure has been proposed by the authors from SHAPE and DMS probing (Xue et al., 2016). The absence of an independently determined folded structure makes fitting the SAXS data particularly difficult, and also complicates interpretation of the data in terms of the standard parameters that are obtained from SAXS experiments. Because this is a first attempt at modeling the folded Bvht RNA, it is imperative that SAXS analyses be clear and comprehensive.

The authors are certainly experts in modeling RNA structures, and this is the strength of the paper. However, the models are only as good as the data, and the representations of the data are not sufficient for this reviewer to be confident in their accuracy. Especially since the authors use their own software to produce the fits (in particular ERNWIN for the ensembles), it is especially important that SAXS data be presented in formats that are becoming standard to the community. The authors should refer to Trewhella et al., 2017 publication guidelines for structural modelling of small-angle scattering data from biomolecules in solution: an update. Acta Cryst. (2017). D73, 710–728.

Specifically, the experimental data should be presented for the Guinier regime (ln(I(q) vs q2) to assess the accuracy of Rg (the authors cite Chen and Pollack, Wiley Interdiscip Rev RNA. 2016, who show the importance of these regions of q). If Bvht is too large to assess linearity at small q, then the authors should discuss the possible errors in determination of Rg in more detail.
They should include Kratky plots (q2*I vs q) to illustrate foldedness or extensions (bell-shaped at low q is typical of folded, flat plateau at high q indicates extended).
They have chosen to report P(r) vs r plots, which is problematic since they have to estimate dmax (Dmax) from their data (see Trewhella's discussion). While such a representation might be more intuitively apparent to some readers, it is the most subjective representation of the data and the most sensitive to over-interpretation. They do not provide error estimates on Dmax in Tables, but refer to the Discussion; the Discussion does not clarify the uncertainties.

Other concerns, in no particular order of importance:
1. The protein CNBP binds to Bvht at the region shown in Figure 3 as the RHT/AGIL site (authors' previous data), where it recognizes an AG-rich sequence. The authors use SAXS to ask if binding of CNBP alters Bvht structure, using full length RNA and fragment 1 (which contains the AGIL site). They also assay for protein binding using EMSA.
They need to show the SAXS data and analysis for CNBP. Certainly the scattering intensity will be much lower than that of the RNA, but since they model the structure (Figures S8 and S10) they should show I(q) vs q, Guinier plots, and Kratky plots. As they discussed in their previous paper (Xue et al., 2016), CNBP has seven CCHC zinc fingers that might participate in RNA binding, but these are not clear in their proposed solution structure (Figure S8; please label the fingers). And since these fingers require Zn2+ to form stable structures, was this ion present in their binding reactions (EMSA) and the SAXS experiments?
At the concentrations of RNA and protein in the SAXS experiments, have the authors demonstrated that there is a 1:1 stoichiometry? There are several other AG-rich sites in Bvht that could possibly be recognized by protein at high enough protein concentrations. In EMSA experiments at the highest protein concentration (Figure 1E), the RNA is stuck in the well, indicating that the complexes have aggregated; certainly the stoichiometry is not 1:1. How does this concentration compare to the protein concentration in SAXS experiments (not stated in Figure 1C)?
In EMSA experiments with Bvht fragments (Table S1), fragment 1 appears to be stuck in the well (based on EtBr intensity). Was there a problem with it aggregating? Since this is the fragment

containing AGIL, it should be bound most efficiently by CNBP, but it seems to be no different than the other fragments. What salts at what concentrations are present for EMSA experiments?
The authors might consider using Stains-all for observing their gels. EtBr is not very sensitive and it leaches out of gels quite quickly.

2. Figure 1 uses ‘s’ as x-axis; use the standard q. State the RNA concentration.

3. Figure 2: D is identical to E.

4. Methods states that an HPLC was inline with SAXS; in the text, this is described as SEC, which is not necessarily equivalent to an HPLC column. What is the correct description? what is the column material?

5. Figure S9. Since the protein is so much smaller than the RNA, and its scattering is much less intense, would you expect to see a difference in global structure of Bvht? Also, if there is a 1:1 stoichiometry, and the AGIL region is exposed to solution and available to protein, would you expect that interaction to influence the rest of the RNA?
In Figure 1A, is there a change in the (interpolated) I(q=0) for the complex? Are there SAXS data for a titration of RNA by protein? If, as the title indicates, this is a study of RNA + protein, then there needs to be a more thorough investigation of the interaction.

6. The discussion is not well organized.

Check English grammar and usage throughout.

Reviewer #2 (Remarks to the Author):

In this manuscript, Kim et al present structural models of the lncRNA Braveheart reconstructed from ensemble SAXS measurements as a function of Mg++ concentration as well as in complex with the CNBP zinc-finger transcription factor. They find that, despite the overall heterogeneity of the structural ensemble, certain trends can be established; Bvht tends towards more compact conformations at elevated Mg++ concentrations, as might be expected. Interestingly, they show that Bvht structure is modular in that the conformational envelopes of non-overlapping subdomains are consistent with subsets of the full-length "structure". Also of note is the finding that CNBP binding leads to a slight compaction without significantly altering the apparent structural ensemble of Bvht. Atomic models are presented that recapitulate the observed SAXS profiles.

This is an interesting work because it essentially proposes that lncRNA functionality is linked to its conformational heterogeneity - that while robust secondary structural elements do exist, the highly flexible regions that link them together potentially enhance binding by protein partners - while also opposing attempts by traditional structural biology techniques to isolate a single dominant 3D structure. These are exciting results, and I think they should be published as it will be of great interest to the larger RNA structural biology community.

I do think the authors could be more strategic in their discussion of the context of their findings. While they spend significant effort touting the advantages of the techniques they have chosen over traditional structural biology techniques, I do not think anyone will dispute that those techniques are clearly unsuited for characterizing lncRNAs. I think novelty of these findings is actually the simplicity of the underlying model, that a lncRNA's conformational ensemble can well-described as rigid, modular subsections concatenated together with flexible single-stranded regions. if this is true it the same methods should hopefully be applicable to other lncRNAs as well. Furthermore, the authors hint that this arrangement may have functional implications for how lncRNAs efficiently interact with protein

binding partners. Would they support the statement that conformational heterogeneity could play a crucial role in lncRNA function? That seems to be what this work is suggesting.

Some minor typos:

Line 190: missing symbols show up as a "square" acsii character in the reviewer PDF

Also in line, 416, 419, 420, 423, 427, 557, 558, 597
And in SI line 52, 56, 67, 93, 95, 97

Reviewer 1:

We are grateful to reviewer 1 for the careful reading of our manuscript and for the many useful and insightful suggestions. We have attempted to address all of the concerns raised. We have made significant changes to the manuscript, adding several new figures, new tables, re-organizing the discussion, clarifying the methodology, and carefully editing the text. We detail these below in a point-by-point response. In the modified manuscript, we high light our changes in green.

General comments from Reviewer 1:

Determination of the tertiary structure of an RNA remains a challenging experimental and computational problem that has no simple solution. Here, the authors attempt to describe the folded structure(s) of a large noncoding RNA, Braveheart (Bvht), from SAXS data and modeling. There is no atomistic structure of Bvht in its tertiary fold, although a secondary structure has been proposed by the authors from SHAPE and DMS probing (Xue et al., 2016). The absence of an independently determined folded structure makes fitting the SAXS data particularly difficult, and also complicates interpretation of the data in terms of the standard parameters that are obtained from SAXS experiments. Because this is a first attempt at modeling the folded Bvht RNA, it is imperative that SAXS analyses be clear and comprehensive.

Authors' Response: We thank the reviewer for this important comment. We agree that SAXS analysis for large RNA systems without prior 3-D structures needs to be especially clear and comprehensive. We followed the necessary steps for SAXS data analysis for our 14 datasets that we have collected using the SEC-SAXS setup at the DIAMOND synchrotron. We have further clarified our analyses in the modified manuscript, adding Guinier plots, Kratky plots, experimental details, and sample concentrations (described in more detail below).

Reviewer 1's main concerns: The authors are certainly experts in modeling RNA structures, and this is the strength of the paper. However, the models are only as good as the data, and the representations of the data are not sufficient for this reviewer to be confident in their accuracy. Especially since the authors use their own software to produce the fits (in particular ERNWIN for the ensembles), it is especially important that SAXS data be presented in formats that are becoming standard to the community. The authors should refer to Trewhella et al., 2017 publication guidelines for structural modelling of small-angle scattering data from biomolecules in solution: an update. Acta Cryst. (2017). D73, 710–728. Specifically, the experimental data should be presented for the Guinier regime (ln(I(q) vs q2) to assess the accuracy of Rg (the authors cite Chen and Pollack, Wiley Interdiscip Rev RNA. 2016, who show the importance of these regions of q). If Bvht is too large to assess linearity at small q, *then the authors should discuss the possible errors in determination of Rg in more detail.* They should include Kratky plots (q2*I vs q) to illustrate foldedness or extensions (bell-shaped at low q is typical of folded, flat plateau at high q indicates extended). They have chosen to report P(r) vs r plots, which is problematic since they have to estimate dmax (Dmax) from their data (see Trewhella's discussion). While such a representation might be more intuitively apparent to some readers, it is the most subjective representation of the data and the most sensitive to over-interpretation. They do not provide error estimates on Dmax in Tables, but refer to the Discussion; the Discussion does not clarify the uncertainties.

Authors' Response: We appreciate this suggestion to add Guinier and Kratky plots as specified in the excellent description of guidelines by Trewhella et al., 2017. While we only report P(r) vs. r in the original manuscript, we now include Guinier and Kratky plots in the revised manuscript, as suggested, to aid in the assessment of accuracy of Rg and to illustrate foldedness, and cite Trewhella et al. accordingly. Thus, for each dataset, we include raw data plots, Guinier plots and Kratky plots (new Supplementary Figures 11 and 12). The *Bvht* Guinier plots display linearity for small q values. Additionally, we have included information on all RNA & protein sequences, sample preparation, purification methods as well as data collection and analysis methods. Thus, we believe that we have reported all crucial information, similar to previous SAXS studies (e.g. Trewhella et al. 2017). The reviewer also points out that we did not provide error estimates on D_{max} in Tables, but referred to this to the discussion. This is an important correction - since D_{max} represents the radius where P(r) approaches to zero, it is not straight forward to estimate errors in this quantity. Thus, we have removed the reference to errors in D_{max}, and explained how we determine Dmax as well as issues regarding D_{max} in the main text. We followed the guidance from Trewhella et al. e.g.

> "This said, it is not correct to simply choose a Dmax that provides a solution that agrees with the Guinier Rg. Rather, the P(r) solution must be independently optimized with the understanding that dmax is an input parameter to the indirect transform selected by the user based on the observed fit of the regularized I(q) corresponding to a given P(r) and how P(r) approaches zero at r = 0 and dmax,"

Thus, we estimate D_{max} when corresponding P(r) approaches zero at r = 0 using a conventional method of estimating D_{max} using GNOM program that has been used previously (e.g., Dzananovic, E. et al., Impact of the structural integrity of the three-way junction of adenovirus VAI RNA on PKR inhibition. PLOS ONE 12, e0186849, 2017; Deo, S. et al. Activation of 2′ 5′-Oligoadenylate Synthetase by Stem Loops at the 5′-End of the West Nile Virus Genome. PLoS ONE 9, e92545, 2014; Bernal, et al., Molecular Organization of Soluble Type III Secretion System Sorting Platform Complexes, J. Mol. Biol., vol. 431 (19), pp. 3787-3803, 2019).

We added Supplementary Table 3 to show that our minimum measured *q* values (near zero) are significantly less than π/D_{max} in all cases, consistent with the requirement discussed in Trewhella et al. that,

> "for a particle with maximum dimension D_{max}, the minimum q value measured should be at most $\sim\pi/D_{max.}$ "

We also note that although high q region contains noise, one can obtain information based on the resolution if there is sufficiently good signal.

In accordance with the comment:

> "The dmax value as independently assessed from the P(r) transform should be consistent with, but not guided by, the known dimensions of the system from complementary techniques. There is an inherent uncertainty in dmax that is difficult to quantify in a rigorous and consistent way,"

we cited relevant AUC study in revision's Methods section as a complementary method.

<u>Other specific concerns raised by reviewer 1</u>:

Reviewer 1's other concern #1a: The protein CNBP binds to Bvht at the region shown in Figure 3 as the RHT/AGIL site (authors' previous data), where it recognizes an AG-rich sequence. The authors use SAXS to ask if binding of CNBP alters Bvht structure, using full length RNA and fragment 1 (which contains the AGIL site). They also assay for protein binding using EMSA. They need to show the SAXS data and analysis for CNBP. Certainly, the scattering intensity will be much lower than that of the RNA, but since they model the structure (Figures S8 and S10) they should show I(q) vs q, Guinier plots, and Kratky plots.

Authors' Response: The reviewer raises an important point that has improved our manuscript and analysis. In order to answer whether CNBP binding alters *Bvht* structure, we reported Fig. 1A and 1C in our original manuscript. In the revised manuscript, we now include SAXS data for CNBP alone, including Guinier and Kratky plots (Supplementary Figure 11 - Small angle X-ray scattering data for CNBP). Additionally, we have provided a Kratky plot demonstrating the change in conformation in *Bvht* upon binding with CNBP (Supplementary Figure 13 - Kratky plots of *Bvht*).

Reviewer 1's other concern #1b: they discussed in their previous paper (Xue et al., 2016), CNBP has seven CCHC zinc fingers that might participate in RNA binding, but these are not clear in their proposed solution structure (Figure S8; please label the fingers).

Authors' Response: The reviewer raises an interesting point that CNBP has seven CCHC zinc fingers and that these may participate in RNA binding. Since little experimental information is available concerning the detailed structure of CNBP, we deleted the CNBP modeling part.

Reviewer 1's other concern #1c. And since these fingers require Zn2+ to form stable structures, was this ion present in their binding reactions (EMSA) and the SAXS experiments?

Authors' Response: The reviewer raises the important question concerning the presence of Zn2+. Yes, we believe that Zinc was present in our experiments. When we expressed CNBP, we used the Luria Broth medium that contains zinc (~170 mg of zinc per kg) and other ions.

Reviewer 1's other concern #1d. At the concentrations of RNA and protein in the SAXS experiments, have the authors demonstrated that there is a 1:1 stoichiometry? There are several other AG-rich sites in Bvht that could possibly be recognized by protein at high enough protein concentrations. In EMSA experiments at the highest protein concentration (Figure 1E), the RNA is stuck in the well, indicating that the complexes have aggregated; certainly, the stoichiometry is not 1:1. How does this concentration compare to the protein concentration in SAXS experiments (not stated in Figure 1C)?

Authors' Response:
The reviewer raises the important point that we did not clearly demonstrate 1:1 stoichiometry between RNA and protein. Our estimation of the equimolar binding of *Bvht* and CNBP is based on the fact that we prepared the complex at 1:1 equimolar ratio of RNA:protein and D_{max} analysis. However, this stoichiometry is suggestive rather than definitive. We are, however, confident our complexes do not aggregate, as evidenced by new Guinier plots of *Bvht*. To clarify this, we have

added Supplementary Figure 11 - Guinier plots of *Bvht*. Here we show (A) Full-length *Bvht* at various magnesium concentrations and the *Bvht*-CNBP complex and (B) *Bvht* sub-regions. Overall, these plots show straight lines in the Guinier regime, which are sound indicators of mono-dispersity (non-aggregation). In the revised version, we also have added Supplementary Table 2, indicating the concentrations of biomolecules we used for SEC-SAXS experiments. As seen from SAXS data, the complex of RNA with protein was monodispersed.

Regarding the EMSA data and Fig. 1E, the data demonstrate that at the highest concentrations, where the stoichiometry of CNBP/*Bvht* is 50:1, the protein and RNA could form high-order oligomers based on non-specific or low-affinity interactions. We initially reported the 4th lane of Fig. 1E to show that at least at this stoichiometry, the RNA-protein complex starts to aggregate significantly. However, in this revision version, we have removed the 4th lane to better focus on the major results of the study (i.e., that we show direct *Bvht*-CNBP binding *in vitro* without any other protein factors) and avoid any misinterpretation.

In summary, for EMSA, the estimated stoichiometries for full-length *Bvht were as follows:*

Lane #	1	2	3
Bvht [μM]	0.05	0.05	0.05
CNBP [μM]	0.00	0.84	1.67
Stoichiometry (CNBP/*Bvht*)	0.0	16.7	33.4

and for fragments of *Bvht:*

Bvht	**Fragment 1**					**Fragment 2**			**Fragment 3**		
Lane #	1	2	3	4	5	6	7	8	9	10	11
Bvht [μM]	0.20	0.20	0.19	0.18	0.16	0.05	0.05	0.05	0.05	0.05	0.05
CNBP [μM]	0.00	0.50	1.00	2.00	4.00	0.00	0.84	1.67	0.00	0.84	1.67
Stoichiometry (CNBP/*Bvht*)	0.0	2.6	5.3	11.1	25.0	0.0	16.7	33.4	0.0	16.7	33.4

Although there are several other AG-rich sites in *Bvht*, as pointed out by the reviewer, our collaborator demonstrated, in the context of cell biology experiments, that the RHT/AGIL region and CNBP interact by CRISPR/Cas9-mediated homology-directed repair (HDR) and protein microarray analysis (Xue, *et al.,* A G-Rich Motif in the lncRNA Braveheart Interacts with a Zinc-Finger Transcription Factor to Specify the Cardiovascular Lineage. *Mol. Cell* **64**, 37–50 (2016)).

Reviewer 1's other concern #1e. In EMSA experiments with Bvht fragments (Table S1), fragment 1 appears to be stuck in the well (based on EtBr intensity). Was there a problem with it aggregating? Since this is the fragment containing AGIL, it should be bound most efficiently by CNBP, but it seems to be no different than the other fragments. What salts at what concentrations are present for EMSA experiments?
The authors might consider using Stains-all for observing their gels. EtBr is not very sensitive, and it leaches out of gels quite quickly.

Authors' Response: The reviewer raises the important concern that, in the EMSA for RNA fragments, fragment 1 may be aggregating. For the case of fragment 1 (Table 1), it is likely that, what appears to be an aggregate in each well is a shadow: the same feature appears in all the lanes,

including lane 1, where no protein was added. In the case of fragment 2, lanes 6-8 show no shadow/aggregation feature. However, lane 9 does show a feature we believe to be aggregation, and we have deleted this lane in this revision. This lane represents the highest concentration of CNBP studied for fragment 2. While slightly greater gel shifts are observed in fragment 1 relative to fragment 2, and for fragment 2 relative to fragment 3, we observe a significantly stronger shift for the full-length RNA relative to each of the fragments. This suggests that the high affinity interaction requires elements found only in the full-length RNA (*e.g.,* the presence of AGIL in combination with a second region of RNA). We emphasize in the revised manuscript that, although fragments of *Bvht* migrated a little as more CNBP was added, their migration changes were not that obvious when compared to the changes in the EMSA of full length *Bvht*.

We have now included a description of our TBE buffer composition (e.g. 45 mM Tris, 45 mM Boric acid, 1 mM EDTA disodium salt, pH 8.3) and magnesium concentration (e.g. 2mM Mg^{2+}) in our EMSA methods section in this revision. We will try stains-all for our future experiments and appreciate this generous advice.

Reviewer 1's other concern #2. Figure 1 uses 's' as x-axis; use the standard q. State the RNA concentration.

Authors' Response: We have updated all the relevant figures.

Reviewer 1's other concern #3: Figure 2: D is identical to E.

Authors' Response: We appreciate this correction. We have updated this section accordingly.

Reviewer 1's other concern #4: Methods states that an HPLC was inline with SAXS; in the text, this is described as SEC, which is not necessarily equivalent to an HPLC column. What is the correct description? what is the column material?

Authors' Response: We appreciate this important request for clarification. DIAMOND has several options, including Size-Exclusion Chromatography (SEC) coupled SAXS with either Superdex or Shodex SEC columns controlled by an Agilent HPLC, with SAXS samples measured through a temperature-controlled capillary. We have updated this section of the manuscript's methods section.

Reviewer 1's other concern #5: Figure S9. Since the protein is so much smaller than the RNA, and its scattering is much less intense, would you expect to see a difference in global structure of Bvht? Also, if there is a 1:1 stoichiometry, and the AGIL region is exposed to solution and available to protein, would you expect that interaction to influence the rest of the RNA?

Authors' Response: The reviewer raises a good point that, due to the relatively small size of the protein compared to the RNA, one might not expect to see a difference in global structure of the RNA. However, if there is a difference in global structure of *Bvht* due to binding of the CNBP protein, then we would be able to observe that difference. The Kratky plot on *Bvht* and *Bvht* bound

with CNBP (last plot of supplementary Fig. 13 A) reflects this observation. The global difference could be due to (i) multiple regions of the RNA simultaneously interacting with the protein, consistent with our observation of a larger gel shift for the full-length RNA relative to the module RNAs, or (ii) the protein binding to a key region of the RNA responsible for global conformational changes. Examples of mechanism (i) occur in the SAM-I riboswitch RNA, where binding of a small molecule (SAM) results in a large conformational change. In the apo case, the RNA samples extended and compact configurations. Upon, SAM binding, conformational capture occurs, where SAM stabilizes closing of the P1 and P3 helices as well as pseudoknot interactions between the loop of helix P2 and the junction between P3 and P4. CNBP binding to the AGIL region of *Bvht* could be similar to the SAM-I riboswitch situation. If this were the case, CNBP binding to the AGIL region may stabilize additional interactions between CNBP and other regions of *Bvht*. It is worth noting that the more significant mobility shifts found in the full-length *Bvht* at intermediate concentrations are not observed in the EMSA experiments where we used *Bvht* fragments (Supplementary Fig. 1), suggesting that there is at least one more higher affinity site formed in this full-length construct. A second possibility would be (ii), where AGIL constitutes a key region of *Bvht* for conformational changes. Examples of mechanism (ii) in other circumstances occur in the ribosome complex. Here, binding of elongation factor G (a single protein, much smaller than the ribosomal complex), results in a large-scale rotation of half of the ribosome (also thought to occur through conformational capture). In addition, binding of small molecules (antibiotics, e.g., viomycin) also result in large conformational changes of the ribosome. Finally, we note that the neck region of the small subunit of the ribosome is responsible for a large and important conformational change called head swivel. The neck consists of double-axel, with two distinct RNA helices enabling the head rotation. If a similar situation operated in *Bvht*, then AGIL could be one of two regions important for triggering large-scale conformational changes. We have modified the discussion regarding the above considerations.

Reviewer 1's other concern #5c: In Figure 1A, is there a change in the (interpolated) I(q=0) for the complex?

Authors' Response: There is only a minor change; however, it is difficult to attribute this change to change in molecular weight. Therefore, we have not commented on I(q=0).

Reviewer 1's other concern #5d: Are there SAXS data for a titration of RNA by protein?

Authors' Response: It is quite difficult to perform titrations with the required amount of sample and the requirement for fresh refolding immediately before SAXS. Furthermore, the SAXS data we have presented for the complex provides information on a monodispersed complex. Therefore, we did not perform titration experiments.

Reviewer 1's other concern #5e: If, as the title indicates, this is a study of RNA + protein, then there needs to be a more thorough investigation of the interaction.

Authors' Response: The reviewer raises an important point: while we have carefully characterized the conformational ensemble of the RNA using SAXS and structural modelling, the characterization of the protein itself is more limited due to the lack of high-resolution data available. We have

shown, however, that the protein does alter the structure of the RNA by both SAXS and EMSA. As this is the first such observation for an epigenetic lncRNA system, to our knowledge, we modified the title to "Zinc-finger protein CNBP alters the 3-D structure of lncRNA Braveheart in solution" in this revised manuscript.

Reviewer 1's other concern #6: The discussion is not well organized. Check English grammar and usage throughout.

Authors' Response: We apologize for the poor organization of the discussion and grammar mistakes. We have attempted to correct all of the grammar mistakes and improved the organization of the discussion. Specifically, we re-organized the discussion section by beginning with a discussion of how the existence of structure in lncRNA systems is a key issue in the field and how our study represents an important first step in this direction. We then emphasize that our study shows that both the RHT/AGIL motif and other structural elements are required for CNBP binding and continue with a discussion of stoichiometry in our study, as suggested by reviewer 1. We discuss the role of CNBP in the cell and also compare our study to studies using similar methodology, but of small RNAs in bacteria. Finally, we emphasize the advantages of our multidisciplinary strategy.

Reviewer 2

We are grateful to reviewer 2 for the careful reading of our manuscript and for the useful and insightful suggestions. We have addressed all of the concerns raised, which included a re-organization of the discussion section and several typographical corrections. In the modified manuscript, we high light our changes in green.

Reviewer 2's overall comments: In this manuscript, Kim et al present structural models of the lncRNA Braveheart reconstructed from ensemble SAXS measurements as a function of Mg++ concentration as well as in complex with the CNBP zinc-finger transcription factor. They find that, despite the overall heterogeneity of the structural ensemble, certain trends can be established; Bvht tends towards more compact conformations at elevated Mg++ concentrations, as might be expected. Interestingly, they show that Bvht structure is modular in that the conformational envelopes of non-overlapping subdomains are consistent with subsets of the full-length "structure". Also, of note is the finding that CNBP binding leads to a slight compaction without significantly altering the apparent structural ensemble of Bvht. Atomic models are presented that recapitulate the observed SAXS profiles.

This is an interesting work because it essentially proposes that lncRNA functionality is linked to its conformational heterogeneity - that while robust secondary structural elements do exist, the highly flexible regions that link them together potentially enhance binding by protein partners - while also opposing attempts by traditional structural biology techniques to isolate a single dominant 3D structure. These are exciting results, and I think they should be published as it will be of great interest to the larger RNA structural biology community.

I do think the authors could be more strategic in their discussion of the context of their findings. While they spend significant effort touting the advantages of the techniques they have chosen over traditional structural biology techniques, I do not think anyone will dispute that those techniques are clearly unsuited for characterizing lncRNAs. I think novelty of these findings is actually the simplicity of the underlying model, that a lncRNA's conformational ensemble can well-described as rigid, modular subsections concatenated together with flexible single-stranded regions. if this is true it the same methods should hopefully be applicable to other lncRNAs as well. Furthermore, the authors hint that this arrangement may have functional implications for how lncRNAs efficiently interact with protein binding partners. Would they support the statement that conformational heterogeneity could play a crucial role in lncRNA function? That seems to be what this work is suggesting.

Authors' Response: We are grateful for the suggestions to improve the discussion section and we have re-organized it as follows. We now begin the discussion section with a discussion of how the existence of structure in lncRNA systems is a key issue in the field and how our study represents an important first step in this direction. We then emphasize that our study shows that both the RHT/AGIL motif and other structural elements are required for CNBP binding and continue with a discussion of stoichiometry in our study, as suggested by reviewer 1. We then discuss the role of CNBP in the cell and also compare our study to studies using similar methodology, but of small RNAs in bacteria. We emphasize the advantages of our multidisciplinary strategy, which was required to produce one of the largest 3-D models of an RNA-only system. Finally, in light of your specific comments that the "…novelty of these findings is actually the simplicity of the underlying model, that a lncRNA's conformational ensemble can well-described as rigid, modular subsections concatenated together with flexible single-stranded regions," "…that this arrangement may have functional implications for how lncRNAs efficiently interact with protein binding partners," and "…conformational heterogeneity could play a crucial role in lncRNA function", we now emphasize these points in the manuscript.

Reviewer 2's specific comments:

Line 190: missing symbols show up as a "square" acsii character in the reviewer PDF

Also, in line, 416, 419, 420, 423, 427, 557, 558, 597
And in SI line 52, 56, 67, 93, 95, 97

Authors' Response :

We have corrected these mistakes.

REVIEWERS' COMMENTS:

Reviewer #1 (Remarks to the Author):

The authors have addressed my concerns. I would like to see the Kratky plots in the manuscript and not in the supplemental material, if that's possible.

Reviewer #2 (Remarks to the Author):

The revised manuscript is much clearer and addresses all of my initial concerns. I think it will be a nice contribution to the field.

Reviewer #1 (Remarks to the Author):

The authors have addressed my concerns. I would like to see the Kratky plots in the manuscript and not in the supplemental material, if that's possible.

➔ Yes, we moved Kratky plots into the main manuscript. We appreciate your thoughtful comment.

Reviewer #2 (Remarks to the Author):

The revised manuscript is much clearer and addresses all of my initial concerns. I think it will be a nice contribution to the field.

➔ We appreciate your considerate comment.
